# High-temperature PTT/CDT coordination nanoplatform realizing exacerbated hypoxia for enhancing hypoxia-activated chemotherapy to overcome tumor drug resistance

**DOI:** 10.1186/s12951-024-02653-8

**Published:** 2024-06-26

**Authors:** Peng Chang, Yingying Guo, Dan Chen, Ke Li, Wei Wang, Zhihua Yang, Jingwen Ma, Yun Zeng, Wenhua Zhan, Yonghua Zhan

**Affiliations:** 1 School of Life Science and Technology, Xidian University and Engineering Research Center of Molecular and Neuro Imaging, Ministry of Education, Xi’an, 710126 PR China; 2 Institute of Analytical Chemistry and Instrument for Life Science, School of Life Science and Technology, Xi’an Jiaotong University, Xi’an, 710049 PR China; 3 Xi’an Key Laboratory for Prevention and Treatment of Common Aging Diseases, Translational and Research Centre for Prevention and Therapy of Chronic Disease, Institute of Basic and Translational Medicine, Xi’an Medical University, Xi’an, 710021 PR China; 4 Department of Radiation Oncology, General Hospital of Ningxia Medical University, Yinchuan, 750004 PR China; 5 Radiology Department, CT and MRI Room, Ninth Hospital of Xi’an, Xi’an, 710054 PR China

**Keywords:** AQ4N, Hypoxia, High-temperature photothermal therapy, Real-time monitoring, Drug resistance

## Abstract

**Background:**

Hypoxia-activated prodrugs present new opportunities for safe and effective tumor drug resistance therapy due to their high selectivity for hypoxic cells. However, the uneven distribution of oxygen in solid tumor and insufficient hypoxia in the tumor microenvironment greatly limit its therapeutic efficacy.

**Results:**

In this paper, a novel AQ4N-Mn(II)@PDA coordination nanoplatform was designed and functionalized with GMBP1 to target drug-resistant tumor cells. Its excellent photothermal conversion efficiency could achieve local high-temperature photothermal therapy in tumors, which could not only effectively exacerbate tumor hypoxia and thus improve the efficacy of hypoxia-activated chemotherapy of AQ4N but also significantly accelerate Mn^2+^-mediated Fenton-like activity to enhance chemodynamic therapy. Moreover, real-time monitoring of blood oxygen saturation through photoacoustic imaging could reflect the hypoxic status of tumors during treatment. Furthermore, synergistic treatment effectively inhibited tumor growth and improved the survival rate of mice bearing orthotopic drug-resistant tumors.

**Conclusions:**

This study not only provided a new idea for PTT combined with hypoxia-activated chemotherapy and CDT for drug-resistant tumors but also explored a vital theory for real-time monitoring of hypoxia during treatment.

**Supplementary Information:**

The online version contains supplementary material available at 10.1186/s12951-024-02653-8.

## Background

Chemotherapy is currently an important means of clinical treatment for tumors [1, 2]. However, more than 90% of patients suffer from multidrug resistance (MDR) to varying degrees during chemotherapy [3], and conventional chemotherapeutic drugs attack tumor cells and normal cells indiscriminately [4]. In view of this, researchers have explored and designed a variety of tumor-specific microenvironment-responsive prodrugs to achieve tumor-specific chemotherapy, among which hypoxia-activated prodrugs have attracted much attention due to their high selectivity for hypoxic cells [5, 6]. AQ4N, a typical hypoxia-activated prodrug approved by the FDA for clinical studies, has weak DNA affinity and is virtually nontoxic under normoxic conditions [7], but under hypoxic conditions, it can be activated by a cytochrome P450-mediated two-electron reduction reaction and converted to the toxic state AQ4 with a high DNA binding affinity [8]. However, our previous study demonstrated that if AQ4N was used alone for chemotherapy, due to the uneven distribution of tumor hypoxia, it would greatly weaken the therapeutic effect of AQ4N or even lead to therapeutic failure [9]. To solve this problem, a common strategy is to combine AQ4N with photodynamic therapy (PDT) to consume oxygen in the tumor tissue to exacerbate hypoxia and thus increase the ability of hypoxia-activated prodrugs to kill tumors [10–12]. However, since PDT itself is strictly dependent on oxygen, it is difficult for PDT to be effective in the hypoxic microenvironment of tumors, and exacerbating hypoxia further limits the efficiency of PDT, resulting in poor therapeutic efficacy of combination therapy [13–16]. Therefore, it is crucial to explore a more rational synergistic strategy based on hypoxia-activated prodrugs.

As a non-oxygen-dependent physical therapy modality, photothermal therapy (PTT) not only provides an effective means for the treatment of MDR tumors, but also effectively prevents PDT from being ineffective under hypoxic conditions [17–20]. Ablation of large solid tumors can be effectively achieved by short-term, high-intensity treatment of tumors with near-infrared (NIR) lasers using photothermal agents [21]. A series of studies have shown that mild-temperature PTT can alleviate tumor hypoxia [22–24]. In contrast, although high-temperature PTT cannot completely ablate tumors, it can cause direct physical damage to the tumor vasculature, leading to vasoconstriction and rupture, resulting in an insufficient oxygen supply to tumors and exacerbating hypoxia in the tumor microenvironment [19, 25, 26]. Therefore, the prospect of combining high-temperature PTT with hypoxia-activated chemotherapy is attractive. Yin et al. developed a 2D core/shell structured mesoporous Silicene@Silica loaded with AQ4N, which was used for synergistic treatment of tumors by using the core of Silicene to generate thermal shocks to exacerbate hypoxia in the tumor microenvironment under NIR-II laser irradiation to enhance hypoxia-activated chemotherapy [26]. However, the limited penetration depth of the NIR laser limits its efficacy in treating deep tumors, and excessive irradiation time with the NIR laser can harm surrounding normal tissues rather than improve treatment outcomes for small and medium-sized tumors. The hypoxia of these small- and medium-sized tumors is often not significant, so high-temperature PTT in combination with hypoxia-activated chemotherapy may not be able to fundamentally cure the tumors [27]. Therefore, overcoming this limitation is particularly important. Interestingly, chemodynamic therapy (CDT), an emerging cancer therapy, can induce severe oxidative stress to kill small- and medium-sized tumors by converting H_2_O_2_ to hydroxyl radicals (·OH) through a Fenton-like reaction [28–31], and the efficiency of ·OH increases significantly with the increase in temperature [32–34]. Therefore, CDT combined with PTT can achieve the effect of “1 + 1 > 2” [35, 36]. Inspired by the above, high-temperature PTT may achieve tumor ablation with dual enhancement of hypoxia-activated chemotherapy and CDT. In addition, determining the hypoxia status of tumors is critical for hypoxia-activated chemotherapy [37]. Our previous studies showed that photoacoustic (PA) imaging can be used to monitor the blood oxygen saturation (*s*O_2_) of tumors in real time, which can indirectly reflect the hypoxic status of tumors [9, 38]. However, no studies related to long-term monitoring of tumor hypoxia during hypoxia-activated chemotherapy have been reported. Therefore, it is significant to construct a nanoplatform that can provide real-time monitoring of the relationship between hypoxia and therapeutic efficacy.

In this paper, we designed a nanoplatform with high photothermal conversion efficiency (PCE). A schematic representation of the synthesis of the nanoplatform and its therapeutic mechanism is shown in Scheme 1. The coordination of AQ4N, Mn^2+^, and dopamine was achieved via a one-pot method, and the polypeptide GMBP1 was crosslinked on the surface of polydopamine (PDA) to obtain a program-triggered nanoplatform named AQ4N-Mn(II)@PDA-GMBP1 (AMPG NPs). When AMPG NPs were enriched in tumors, they would rapidly be heated under laser irradiation. High temperature exacerbated the degree of hypoxia in the tumor microenvironment, accelerating the reduction of AQ4N to toxic AQ4. Meanwhile, the high temperature accelerated the disintegration of the AMPG NPs to release Mn^2+^ and significantly improved the efficiency of the conversion of endogenous H_2_O_2_ to ·OH. Moreover, the AMPG NPs had excellent PA and magnetic resonance (MR) imaging properties, and we evaluated the hypoxia status of the tumors during treatment through PA imaging and HIF-1α immunohistochemistry (IHC). Notably, in vivo anti-tumor experiments further demonstrated that mild-temperature PTT/AQ4N/CDT was unable to effectively kill tumor cells. In contrast, high-temperature PTT/hypoxia-activated chemotherapy/CDT demonstrated a significant synergistic anti-tumor effect in both tumor-bearing mice and orthotopic tumor models in mice. In summary, this study not only provided a new idea for PTT combined with hypoxia-activated chemotherapy and CDT for drug-resistant tumors but also explored a vital theory for real-time monitoring of hypoxia during treatment.

**Sch. 1:**
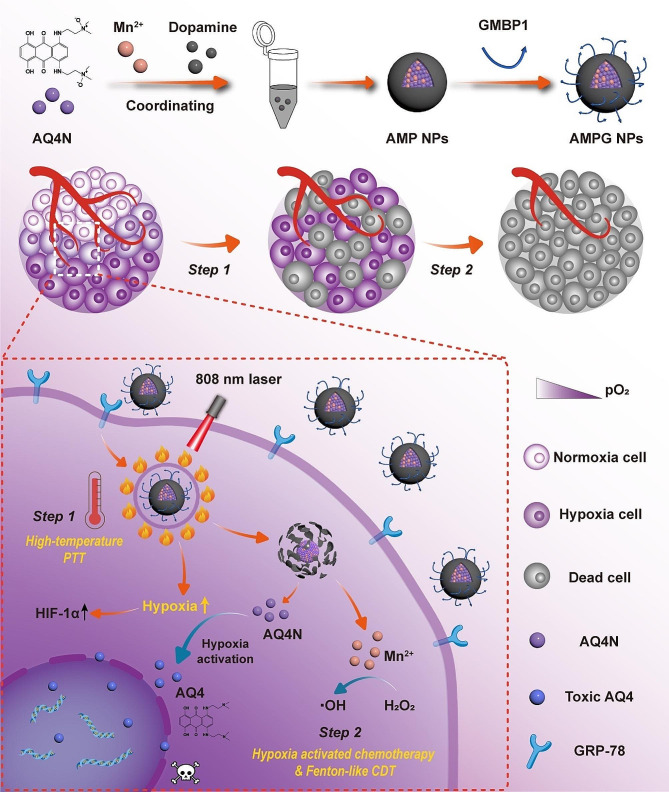
Schematic illustration of AMPG NP synthesis and high-temperature PTT dual-enhanced hypoxia-activated chemotherapy and CDT

## Methods

### Synthesis of AMPG NPs

Synthesis of AQ4N-Mn(II)@PDA NPs (AMP NPs): 20 µL of AQ4N solution (10 mM), 40 µL of MnAc_2_ (20 mM), 200 µL of dopamine hydrochloride solution (1 mg mL^-1^), and 740 µL of Tris-HCl were added to a 1.5 mL centrifuge tube and the final pH of the solution was adjusted to 8.5; the reaction mixture was placed in a thermostatic mixer at room temperature for 24 h. The precipitate (AMP NPs) was collected by centrifugation at 14,600 × g for 10 min and redispersed in PBS.

Synthesis of AMPG NPs: The polypeptide GMBP1 was modified to generate amino groups at both ends. Then, 500 µL of AMP NPs and 500 µL of 0.2 mg mL^-1^ GMBP1 were added to a 1.5 mL tube and reacted in a thermostatic mixer at room temperature for 12 h. The uncoupled GMBP1 was then removed using a PD-10 desalting column to obtain AMPG NPs.

### Characterization of AMPG NPs

The morphology of AMPG NPs was investigated using transmission electron microscopy (TEM) and analyzed elementally using X-ray energy-dispersive spectroscopy (EDS). The elemental and valence analyses of AMPG NPs were performed using X-ray photoelectron spectroscopy (XPS). The hydrated particle size and surface potential of AMP NPs and AMPG NPs were determined using a Malvern dynamic light scattering (DLS) particle size analyzer. The characteristic peaks of the UV-Vis-NIR spectra of AQ4N, GMBP1, AQ4N/Mn^2+^, AMP NPs, and AMPG NPs were measured using a UV-Vis-NIR spectrophotometer. The chemical functional groups in AMP NPs and AMPG NPs were determined using Fourier transform infrared (FTIR) spectroscopy. The in vitro stability of the nanoplatform was assessed by measuring the hydrated particle size of AMP NPs and AMPG NPs in PBS and 10% fetal bovine serum (FBS) solution.

### Release of AQ4N from AMPG NPs

The standard curve of AQ4N was established by preparing a 10 mM solution of AQ4N and diluting it to 0.02, 0.04, 0.06, 0.08, and 0.1 mM with deionized water (DI water) and then measuring the absorbance at 665 nm using a UV-Vis spectrophotometer. In the final step of AMP NPs synthesis, the concentration of free AQ4N in the supernatant was quantified by measuring the UV-Vis absorbance at 665 nm of the supernatant after high-speed centrifugation to calculate the drug encapsulation efficiency (DEE) and drug loading content (DLC). The DEE and DLC of AMPG NPs were calculated from equations (S1) and (S2). The in vitro release of free AQ4N, AQ4N/Mn^2+^, and AMPG NPs was determined by using dialysis. Then, 500 µL of each sample solution was added to the dialysis tube as the internal solution, and the external solution was 10 mL of DI water and incubated at 37 °C. The concentration of AQ4N in the external solution was quantified at 1, 3, 6, 12, 24, and 48 h to determine the cumulative release profile.

### PCE of AMPG NPs

In vitro photothermal performance assessment was achieved by adding 1 mL of sample to a 1.5 mL centrifuge tube, and the fiber-optic collimator was fixed vertically approximately 1 cm above the centrifuge tube. The photothermal performance of different samples was investigated by exposing PBS, AQ4N/Mn^2+^, PDA, and AMPG NPs to an 808 nm laser at 2.0 W cm^-2^, and the temperature was recorded for 5 min at intervals of 30 s using an NIR thermal camera. The photothermal properties of the nanoplatform at different concentrations and laser powers were also investigated using the above methods. Moreover, AMPG NPs were heated with an 808 nm laser in a continuous cycle, and the temperature increased for 5 min followed by cooling for 5 min in each cycle. The PCE of AMPG NPs was calculated from equation (S3). In vivo photothermal performance was evaluated by injecting 200 µL of PBS, AMP NPs, and AMPG NPs into the tumor-bearing mice, and after 3 h, the tumor areas of the mice were irradiated with an 808 nm laser at 2.0 W cm^-2^ for 5 min. Changes in the temperature of the tumor sites before and after irradiation were recorded with an NIR thermal camera.

#### In vitro PA and MR imaging of AMPG NPs

The PA imaging properties of the nanoplatform were analyzed using the MSOT system. An agar phantom was created, and PA imaging was performed using different concentrations of AQ4N (0.0125, 0.025, 0.05, 0.1, and 0.2 mM), the AMPG NPs encapsulated different concentrations of AQ4N (0.0125, 0.025, 0.05, 0.1 and 0.2 mM), and different concentrations of dopamine (0.00625, 0.0125, 0.025, 0.05, and 0.1 mg mL^-1^) for synthesizing PDA NPs were tested in PA imaging.

The MR imaging performance of AMPG NPs was tested using a 0.5T small animal MR imaging scanner. AMPG NPs were diluted into five concentrations (Mn^2+^ concentrations: 0.05, 0.1, 0.2, 0.4, and 0.8 mM), and MnAc_2_ solutions corresponding to Mn^2+^ concentrations were prepared as controls. r_1_ values were calculated by a linear fit of 1/T_1_ (s^-1^) to the sample concentrations (mM).

### Mn^2+^-mediated Fenton-like reactions

The effects of Fenton-like reactions were evaluated using methylene blue (MB) as the target degradant. The concentrations of MB and NaHCO_3_ were fixed at 10 µg mL^-1^ and 25 mM, respectively. The concentration of H_2_O_2_ was first fixed at 8 mM to assess the effect of different concentrations of MnAc_2_ and AMPG NPs (Mn^2+^: 0.00625, 0.0125, 0.025, 0.05, and 0.1 mM). Then, MnAc_2_ and AMPG NPs were fixed at 0.1 mM (Mn^2+^) to assess the Fenton-like activity under different concentrations of H_2_O_2_ (0.5, 1, 2, 4, and 8 mM). All the above reactions were performed at 37 °C for 30 min, the color change of the solution was recorded, and the absorbance was measured at 665 nm at the end of the reaction. To verify that high temperature can significantly increase the efficiency of ·OH generation, the concentrations of MB, NaHCO_3_, H_2_O_2_, and Mn^2+^ were fixed at 10 µg mL^-1^, 25 mM, 8 mM, and 5 µM, respectively. The two groups were reacted at 50 °C and 25 °C for 30 min, the color change of the solution was recorded at the end of the reaction and the absorbance at 665 nm was measured to determine ·OH generation at different temperatures.

## In vitro cell culture studies

### In vitro cell viability

SGC-7901/ADR cells were cultured in DMEM supplemented with 10% FBS and incubated in a 37 °C and 5% CO_2_ incubator. The cytotoxicity of AQ4N was studied under normoxic and hypoxic conditions. When the cells were attached to a 96-well plate and grown to occupy the bottom of the wells to approximately 80%, the medium was replaced with fresh medium containing different concentrations of AQ4N (0, 2.22, 6.67, 20, 60, and 180 µM), and the cells in the two groups were incubated for 24 h, Then, 10 µL of CCK-8 was added to each well and incubated for 2 h. The absorbance of each well at 450 nm was measured using a microplate reader to calculate the cell viability and IC_50_ of AQ4N. The cytotoxicity of AMPG NPs containing different concentrations of AQ4N (0, 1.23, 3.70, 11.1, 33.3, and 100 µM) in a hypoxic environment was determined following the same method. To study the cytotoxicity of the nanoplatform under laser irradiation, 100 µL of fresh medium containing PBS, PDA, AQ4N, and AMPG NPs was added to each well, and the plates were incubated in a hypoxic incubator for 24 h. After incubation for 12 h, the cells in the laser irradiation groups were irradiated with an 808 nm laser at 2.0 W cm^-2^. The cell viability of each group was calculated.

Calcein AM/PI fluorescence di-staining was used to distinguish living and dead cells. When approximately 80% of the cells reached the bottom of wells, the old medium was removed, and the cells were washed with PBS. Then, 1 mL of serum-free medium containing PBS, PDA, AQ4N, and AMPG NPs was added, and the cells were incubated in a hypoxic incubator for 24 h. After 12 h, the cells in the laser irradiation groups were irradiated with an 808 nm laser at 2.0 W cm^-2^. Living cells were stained with Calcein AM (green fluorescence, Ex/Em: 494/517 nm), and dead cells were stained with PI (red fluorescence, Ex/Em: 535/617 nm). After 30 min of staining, the cells were rinsed with PBS and then visualized and analyzed using a fluorescence microscopy.

### Cell uptake and cell affinity

When the cells had grown to occupy the bottom of the wells to approximately 70%, the old medium was removed, the cells were washed three times with PBS, 1 mL of serum-free medium containing AMP NPs was added, and the cells were incubated for 1, 2, 4, 8, and 12 h, respectively. At each time point, the old medium was removed, and the cells were washed three times with PBS, fixed with 4% paraformaldehyde for 5 min, and washed three times with PBS. After 100 µL of DAPI staining and three washes with PBS, the cell uptake of AMP NPs was observed using a confocal laser scanning microscopy (CLSM), in which DAPI emitted blue fluorescence (Ex/Em: 405/430–470 nm), while AQ4N emitted red fluorescence (Ex/Em: 612/630–700 nm).

Drug-resistant and non-resistant tumor cells were chosen and divided into 3 groups: targeting, non-targeting, and blocking groups (blocking groups: drug-resistant tumor cells were first incubated with GMBP1 and then incubated with AMPG NPs). The cell affinity of AMPG NPs was observed using the CLSM, and the fluorescence intensity was quantified using ImageJ.

### In vitro ROS detection

ROS levels were detected using a DCFH-DA fluorescent probe. Tumor cells were incubated for 4 h in medium supplemented with different concentrations (Mn^2+^: 0, 100, 200, 400, and 800 µM) of AMPG NPs. The medium was removed, the cells were gently rinsed three times with PBS, and fresh medium containing DCFH-DA (10 µM) was added to the cells which were incubated for another 20 min. Then, the old medium was removed, and the cells were gently rinsed three times with PBS and subsequently fixed with 4% paraformaldehyde for 5 min. Finally, the cells were observed using the CLSM, and the fluorescence intensity of the cells was quantified using ImageJ.

### Flow cytometry

When the cells had grown to occupy the bottom of the wells to approximately 80%, the old medium was removed, and the cells were washed with PBS. Then, 100 µL of fresh medium containing PBS, PDA, AQ4N, and AMPG NPs was added to each well, and the cells were incubated in a hypoxic incubator for 24 h. In the laser irradiation group, the cells were irradiated with an 808 nm laser at 2.0 W cm^-2^ for 5 min at the 12-h time point and then put into the incubator for further incubation. At the end of incubation, the cells were digested and collected in centrifuge tubes, centrifuged at 1,000 × g for 5 min, washed twice, and resuspended in PBS. Finally, the cells were analyzed for apoptosis using the Annexin V-FITC/PI Apoptosis Detection Kit, and the flow cytometry results were analyzed.

### In vivo PA and MR imaging of AMPG NPs

For PA imaging, tumor-bearing mice were divided into three groups and injected intravenously with 200 µL of saline, AMP NPs, and AMPG NPs (doses: 0.2 mM AQ4N; 0.8 mM Mn^2+^), and the imaging coupling agent was uniformly applied to the tumor area before and 1, 3, 6, 12, and 24 h after injection. Then, the mice were fixed in the tank for PA imaging, and the images of the tumor area were quantified using MSOT. MR imaging was performed after injecting 200 µL of saline, AMP NPs, and AMPG NPs intravenously into the mice (doses: 0.2 mM AQ4N; 0.8 mM Mn^2+^). MR images of the tumors were captured before and 6 h after injection, and the gray values of the tumor area were quantified using ImageJ.

### In vivo tumor hypoxia monitoring

When the tumor size reached 300–400 mm^3^, the tumor-bearing mice were divided into 5 groups, and the mice in each group were injected with 200 µL of saline, AQ4N, AMPG NPs, AMPG NPs with mild-temperature PTT, and AMPG NPs with high-temperature PTT (AQ4N dose: 0.2 mM). Six hours after injection, the mice in the mild-temperature PTT and high-temperature PTT groups were irradiated with an 808 nm laser at 1.5 W cm^-2^ and 2.0 W cm^-2^, respectively. After 24 h, the mice were euthanized, and the tumors were harvested, fixed in 4% paraformaldehyde and stored at 4 °C for further sectioning, staining, and analysis. The tumor volume was calculated from equation (S4).

When the tumor size of the tumor-bearing mice reached 300–400 mm^3^, the mice were divided into 6 groups (*n* = 3), and the mice in each group were injected with 200 µL of saline, AQ4N, AMPG NPs, AMPG NPs with mild-temperature PTT, and AMPG NPs with high-temperature PTT on days 1, 4, 7, and 10, respectively (AQ4N dose: 0.2 mM), in which the tumors were irradiated 6 h after injection. PA imaging was used to capture Hb and HbO_2_ signals in the tumor area of mice on days 0, 3, 6, 9, and 12. The images were quantified and analyzed using the MSOT system by the following Eq. (1) for *s*O_2_:

$$s\text{O}_{2}\,(\%) =\frac{{\text{HbO}}_{2}}{\text{Hb}+{\text{HbO}}_{2}} \times 100\%\,(1)$$

At the end of treatment, the mice were euthanized, and the tumors were harvested, fixed in 4% paraformaldehyde, and stored at 4 °C for further sectioning, staining, and analysis.

### In vivo synergistic PTT/hypoxia-activated chemotherapy/CDT

The tumor signals of the orthotopic tumor model mice were monitored using an IVIS small animal imaging system. The mice were divided into 8 groups (*n* = 3): saline, Laser (L), AQ4N, AQ4N + L, AMP NPs, AMP + L, AMPG NPs, and AMPG NPs + L (AQ4N dose: 0.2 mM). Treatment was performed on a three-day cycle, in which bioluminescence imaging was performed to characterize tumor changes on the first day of each cycle; on the second day, 200 µL of sample from each group was injected intravenously, and the mice in the laser irradiation group were irradiated with an 808 nm laser at 2.0 W cm^-2^ for 5 min at 6 h post-injection (on the third day). The entire treatment cycle lasted 21 days. The body weights and survival rates of the mice in each group were recorded during the treatment period. After 21 days, the mice were euthanized, the tumors were dissected, the tumor sizes and weights were measured, and the tumors were fixed in 4% paraformaldehyde and stored at 4 °C for further sectioning, staining, and analysis.

### Staining of tissue sections

Organs and tumors were embedded in paraffin blocks, sectioned at a thickness of 5 µm using a microtome, and stained for microscopic analysis with hematoxylin and eosin stain (H&E). The antigenically repaired sections were then incubated with primary antibody (mouse monoclonal antibody, 1:200 dilution) conjugated to HIF-1α for IHC staining. After the sections were incubated with the secondary antibody and horseradish peroxidase, a brown insoluble DAB (3,3’-diaminobenzidine) precipitate was generated, and photographs were captured. Terminal deoxynucleotidyl transferase dUTP-biotin nick end labeling (TUNEL) was used to visualize apoptosis in tumor tissue sections.

### Statistical analysis

The data were obtained and represented at least three times as mean ± SD. Two or more groups were compared using one-way ANOVA with a Newman-Keuls test as a post-hoc analysis (R software, version 4.1.2). No significant differences (*n.s.*), *P* < 0.05 (*), *P* < 0.01 (**), and *P* < 0.001 (***) were indicated.

### Results and discussion

#### Synthesis and characterization of AMPG NPs

**Fig. 1:**
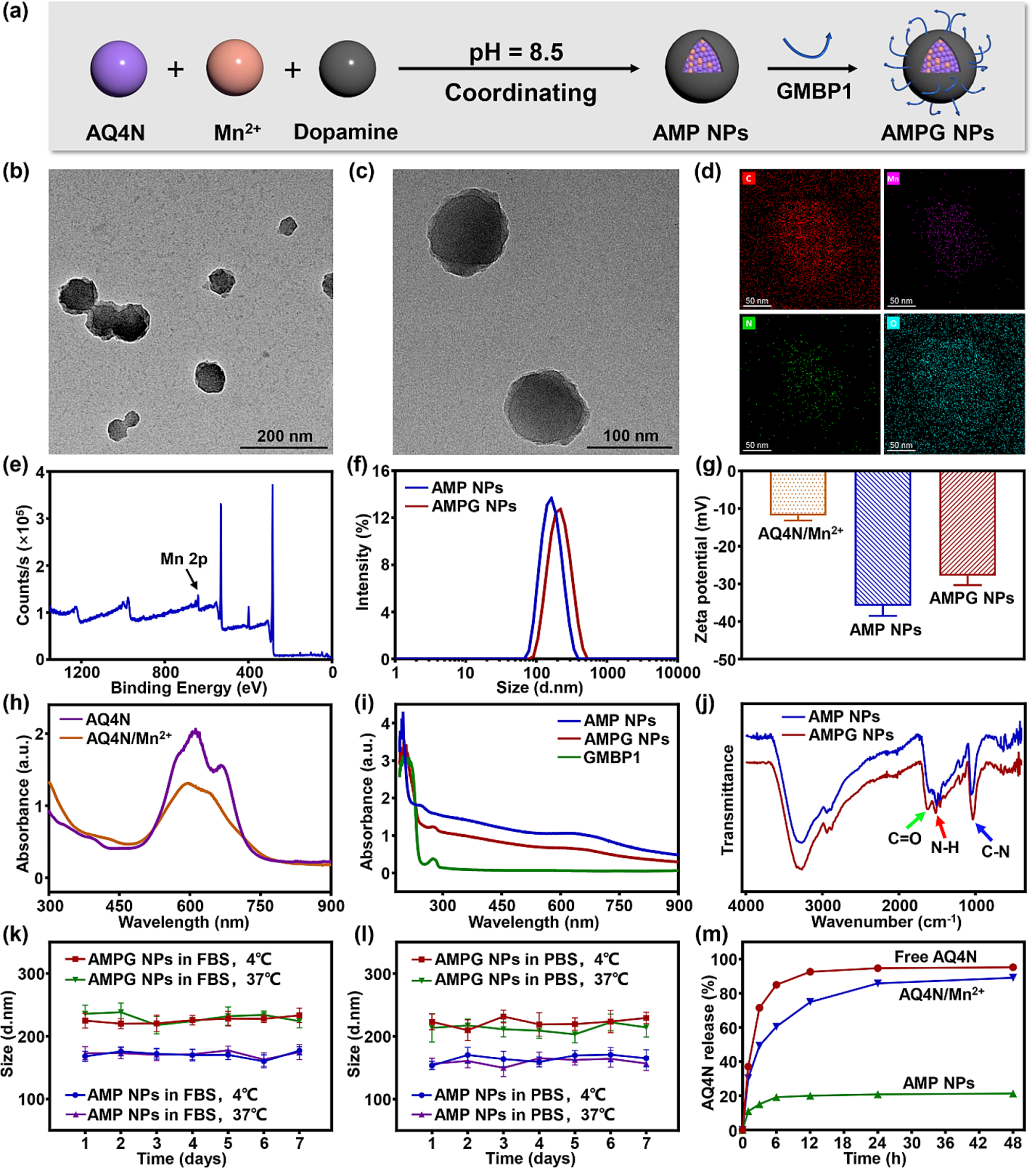
Characterizations of AMPG NPs. **a**) Synthesis of AMPG NPs. **b**) TEM image of AMPG NPs; scale bar = 200 nm. **c**) Zoomed TEM image of AMPG NPs; scale bar = 100 nm. **d**) EDS mapping and elemental analysis of AMPG NPs; scale bar = 50 nm. **e**) XPS spectrum of AMPG NPs. **f**) DLS analysis to characterize the size distribution of AMP NPs and AMPG NPs. **g**) DLS analysis to characterize the surface Zeta potential of AMP NPs and AMPG NPs. **h**) UV-Vis-NIR spectra of AQ4N and AQ4N/Mn^2+^. **i**) UV-Vis-NIR spectra of GMBP1, AMP NPs, and AMPG NPs. **j**) FTIR spectra of AMP NPs and AMPG NPs. **k**) Size distributions of AMP NPs and AMPG NPs in 10% FBS in a 7-day stability test. **l**) Size distributions of AMP NPs and AMPG NPs in PBS in a 7-day stability test. **m**) Free AQ4N release and AQ4N release from AQ4N/Mn^2+^ and AMPG NPs

The scheme of AMPG NP synthesis is shown in Fig. 1a, where the coordination of AQ4N, Mn^2+^, and dopamine was used in a one-pot method. As shown in Fig. S1, to facilitate the grafting of the targeting peptide GMBP1 to PDA, GMBP1 was modified with amino groups at both ends [39]. AMPG NPs with diameters of approximately 100 nm were successfully synthesized spherically, as illustrated in the TEM images in Fig. 1b and c. As shown in Fig. S2a, four different samples (Mn^2+^, AQ4N, PDA, and AMPG NPs) were prepared. As seen from the photographs, the PDA solution was brown, but surprisingly, the solution became almost completely black after the addition of AQ4N/Mn^2+^, which was confirmed by the UV-Vis-NIR absorbance in Fig. S2b, where the UV-Vis-NIR absorbance of AMPG NPs at 808 nm increased compared with that of PDA, indicating that AMPG NPs were able to efficiently absorb all wavelengths of visible-NIR light, thus significantly increasing the PCE, which facilitated the subsequent realization of in vivo high-temperature PTT. As shown in Fig. 1d and e, the elemental mapping by EDS and XPS analysis of divalent Mn confirmed the successful synthesis of the nanoplatform, and the EDS mapping is shown in Fig. S3.

The hydrated particle size distributions of AMP NPs and AMPG NPs are shown in Fig. 1f, which were 154.6 ± 6.4 nm and 201.3 ± 10.2 nm, respectively, wherein the addition of GMBP1 slightly increased the hydrated particle size of the nanoplatform. As shown in Fig. 1g, the zeta potential of AQ4N/Mn^2+^ was − 11.8 ± 1.3 mV, which was negatively charged, and after co-coordinating with PDA to form AMP NPs, the zeta potential further decreased to -35.8 ± 2.7 mV due to the large number of hydroxyl groups on the surface of PDA; whereas the zeta potential of AMPG NPs, due to the small amount of amino groups that were grafted onto the PDA surface of GMBP1, increased to -27.8 ± 2.5 mV, which indicated successful synthesis of AMPG NPs. Figure 1h and i show the UV-Vis-NIR absorbance peaks of AQ4N, AQ4N/Mn^2+^, GMBP1, AMP NPs, and AMPG NPs. The characteristic UV-Vis peaks of GMBP1 and AQ4N were at 280 and 665 nm, respectively. AMP NPs and AMPG NPs showed the same absorbance peaks as did AQ4N, indicating that AQ4N was successfully encapsulated in PDA. In addition, AMPG NPs showed the same absorbance peaks as GMBP1, indicating that GMBP1 was successfully grafted onto the surface of PDA. Figure 1j shows the differences in the FTIR spectra of AMP NPs and AMPG NPs and the C-N stretching vibration peak at 1033 cm^-1^ as well as the N-H bending vibration peak at 1516 cm^-1^ for the characteristic peaks of PDA, indicating that PDA was successfully polymerized. When GMBP1 was grafted onto PDA, the characteristic peak at 1630 cm^-1^ was attributed to the C = O stretching vibration and N-H bending vibration of the polypeptides, indicating the successful synthesis of AMPG NPs. Figure 1k and l show the hydrated particle size distributions of AMP NPs and AMPG NPs in 10% FBS and PBS, respectively, over 7 days. The size distributions of AMP NPs and AMPG NPs were approximately 150 nm and 200 nm, respectively, indicating that both of them had high stability in different media.

### The controlled release of AQ4N from AMPG NPs

The regression curve of AQ4N is shown in Fig. S4, and the regression Eq. (2) is:

$$Y\hspace{0.17em}=\hspace{0.17em}5.605X\hspace{0.17em}+\hspace{0.17em}0.05830 ({R\hspace{0.17em}}^{2}=\hspace{0.17em}0.9995) \left(2\right)$$

After the successful synthesis of AMPG NPs, free AQ4N was separated from the nanoplatform by centrifugation, the DEE of AQ4N was calculated to be 78.67%, and the DLC was 19.39% from the measurement of the UV-Vis of the supernatant. The in vitro drug release profiles of free AQ4N, AQ4N/Mn^2+^, and AMPG NPs solutions are shown in Fig. 1m. Free AQ4N had the fastest drug release rate, with approximately 70% released at 3 h and more than 90% released after 12 h. Compared with free AQ4N, AQ4N/Mn^2+^ slightly slowed the release rate of AQ4N, but it still released more than 80% after 48 h. This was due to the weak coordination effect of AQ4N and Mn^2+^ and poor stability. However, in the AMPG NPs group, the release rate of AQ4N after 48 h was only 21% due to the formation of a strong coordinating polymer structure between PDA and AQ4N/Mn^2+^, which significantly enhanced the in vitro controlled release of AQ4N. In addition, the pH-responsiveness and photothermal effect of PDA could effectively cleave PDA after targeted administration, thus realizing quick and precise AQ4N release.

#### In vitro photothermal effect of AMPG NPs

**Fig. 2:**
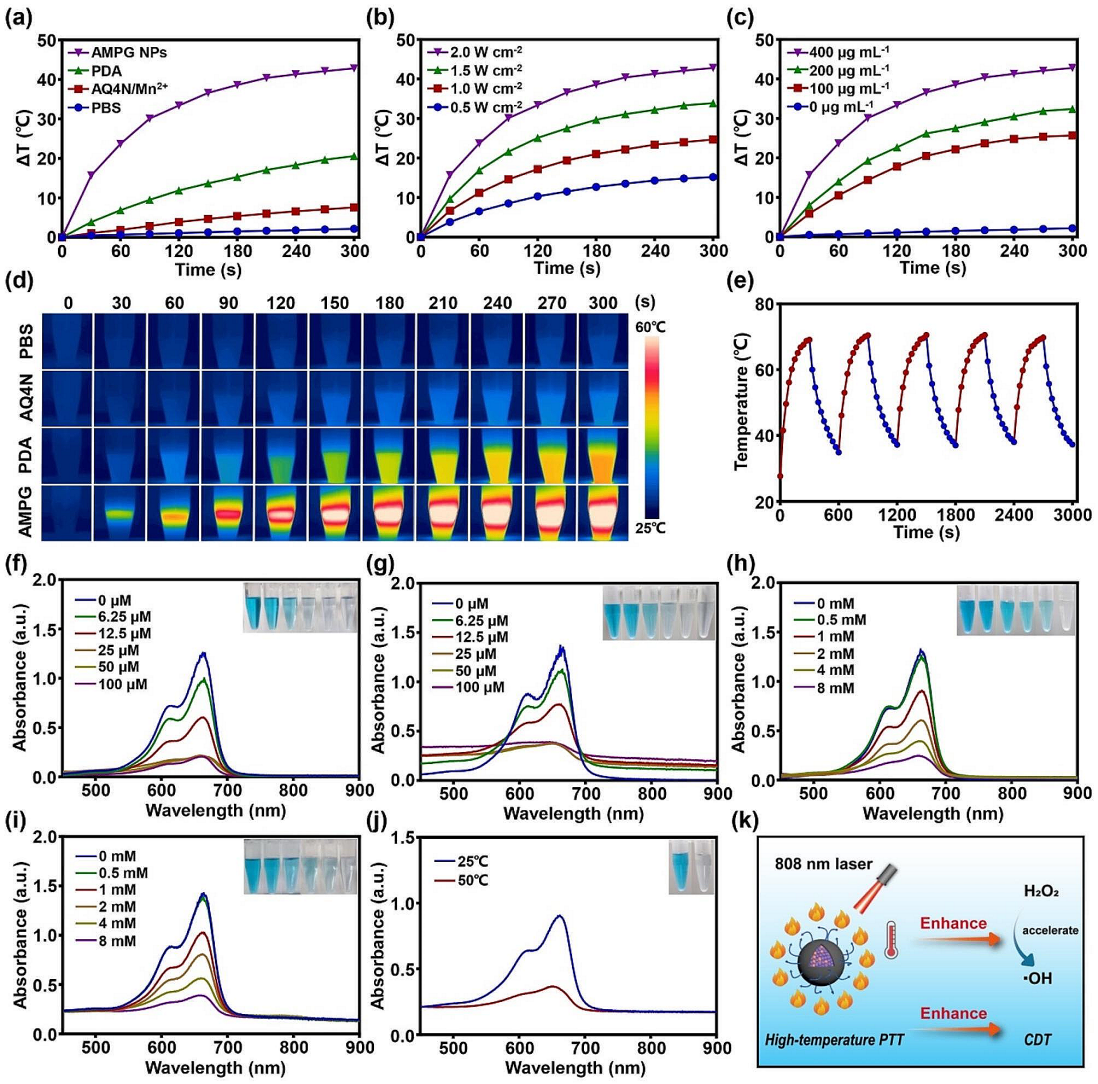
In vitro photothermal conversion and chemodynamic activity of AMPG NPs. **a**) The effect of 808 nm laser irradiation on the temperature rises of PBS, AQ4N/Mn^2+^, PDA, and AMPG NPs. **b**) The effect of 808 nm laser irradiation on the temperature increases of AMPG NPs at 0.5, 1.0, 1.5, and 2.0 W cm^-2^. **c**) The effect of 808 nm laser irradiation at concentrations of 0, 100, 200, and 400 µg mL^-1^ on the temperature rises of AMPG NPs. **d**) NIR thermal images of PBS, AQ4N/Mn^2+^, PDA, and AMPG NPs during the photothermal conversion process. **e**) Heating cycles of AMPG NPs using an 808 nm laser. UV-Vis-NIR spectra and photographs of MB degradation **f**) under different concentrations of MnAc_2_ and 8 mM H_2_O_2_, **g**) under different concentrations of AMPG NPs and 8 mM H_2_O_2_, **h**) under different concentrations of H_2_O_2_ and 100 µM MnAc_2_, **i**) under different concentrations of H_2_O_2_ and AMPG NPs (Mn^2+^: 100 µM), and **j**) at different temperatures under 8 mM H_2_O_2_ and AMPG NPs (Mn^2+^: 100 µM). **k**) Schematic illustration of accelerated ·OH generation at a high temperature

The PCE of the nanoplatform is crucial for achieving localized high-temperature PTT in tumors, as is the efficacy of hypoxia-activated chemotherapy and CDT. Therefore, we investigated the in vitro photothermal effect of AMPG NPs. Figure 2a shows the photothermal profiles of PBS, AQ4N/Mn^2+^, PDA, and AMPG NPs under 808 nm laser irradiation at 2.0 W cm^-2^, and the temperatures of PBS, AQ4N, and PDA increased by 2.2, 7.6, and 20.6 °C, respectively, while the temperature of AMPG NPs increased by 42.8 °C. Figure 2b and c show the photothermal profiles of AMPG NPs under laser irradiation with different powers (0.5, 1.0, 1.5, and 2.0 W cm^-2^) and the photothermal profiles of AMPG NPs with different concentrations (0, 100, 200, and 400 µg mL^-1^) under laser irradiation at 2.0 W cm^-2^, respectively, and the NIR thermal images are shown in Fig. 2d. In addition, the PCE of AMPG NPs was calculated to be 46.79%, which was comparable to that of nanoplatform with metal-PDA polymer as the photothermal agent [27]. All these studies show that AMPG NPs have excellent photothermal performance. The results indicate that when the concentration of AMPG NPs and the laser power increased, the solution temperature also increased. Figure 2e shows the heating cycle curves after 808 nm laser irradiation, indicating that AMPG NPs exhibited strong photothermal stability after multiple heating cycles.

#### In vitro chemodynamic of AMPG NPs

To verify that the nanoplatform has good Fenton-like activity for realizing efficient CDT in vivo, we performed in vitro chemodynamic studies of AMPG NPs. Figure 2f and g show the Fenton-like activity of MnAc_2_ and AMPG NPs with different concentrations of Mn^2+^. Figure 2h and i show the Fenton-like activity of MnAc_2_ and AMPG NPs with different concentrations of H_2_O_2_. The Fenton-like activities of MnAc_2_ and AMPG NPs increased significantly with increasing Mn^2+^ concentration. In addition, to further elucidate the effect of temperature on ·OH generation efficiency, we evaluated the Fenton-like activity of AMPG NPs at different temperatures, as shown in Fig. 2j. The absorbance at 665 nm at the end of the reaction at high temperature was significantly lower than that at room temperature, indicating that high temperature could significantly increase the efficiency of ·OH generation; a schematic illustration is shown in Fig. 2k. These results indicate that AMPG NPs have good in vitro chemodynamic activity and have potential for in vivo CDT.

#### In vitro PA and MR imaging of AMPG NPs

**Fig. 3:**
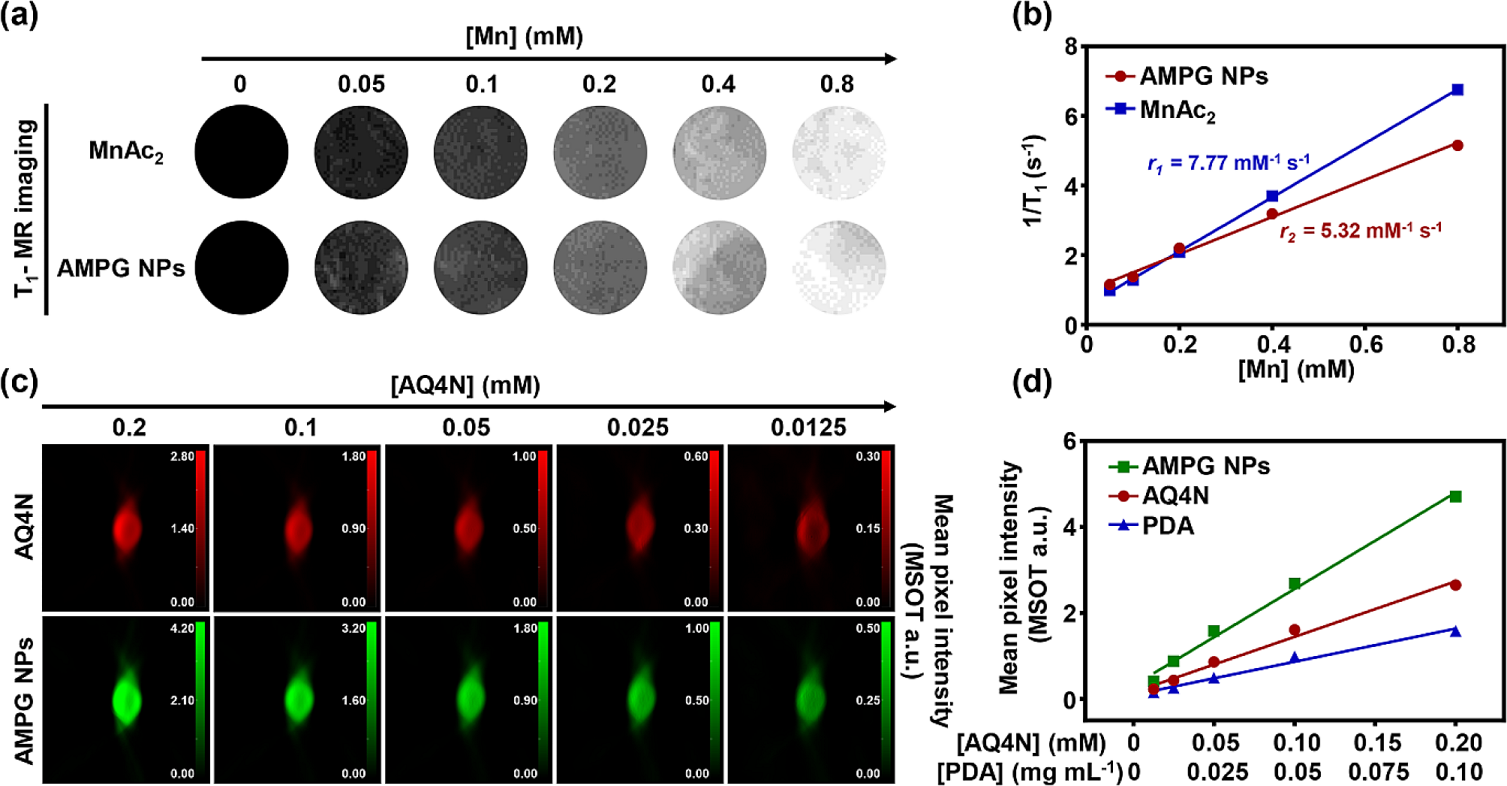
In vitro PA and MR imaging of AMPG NPs. **a**) In vitro T_1_-weighted MR images of MnAc_2_ and AMPG NPs. **b**) Linear regression of the T_1_ relaxation rate curves of MnAc_2_ and AMPG NPs. **c**) PA images of different concentrations of AQ4N and AMPG NPs (0.0125, 0.025, 0.05, 0.1, and 0.2 mM) in the phantom. **d**) Linear regression of the concentrations of AQ4N and PA imaging intensities

To test the MR imaging contrast ability of AMPG NPs, we used Mn^2+^ with AMPG NPs for comparison. As shown in Fig. 3a, the T_1_-weighted MR imaging signals of both the MnAc_2_ and AMPG NPs significantly increased with increasing Mn^2+^ concentration. As shown in Fig. 3b, linear fitting was performed with 1/T_1_ as the vertical coordinate and Mn^2+^ concentration as the horizontal coordinate, and the relaxation rates of MnAc_2_ and AMPG NPs were calculated to be 7.77 mM^-1^·s^-1^ and 5.32 mM^-1^·s^-1^, respectively, indicating that AMPG NPs had good MR imaging visualization ability. The r_1_ values of AMPG NPs were lower than those of MnAc_2_ because PDA encapsulated Mn^2+^, reducing the relaxation effect of Mn^2+^.

The In vitro PA imaging properties of AQ4N, PDA, and AMPG NPs are shown in Fig. 3c and S5, and the PA signals of the different materials were positively correlated with the concentrations of AQ4N and PDA. Meanwhile, AMPG NPs were able to absorb all wavelengths of visible-NIR light efficiently, showing more powerful PA imaging performance than PDA and AQ4N. According to the linear fit of the mean pixel intensity against the concentrations of AQ4N, PDA, and AMPG NPs, AMPG NPs significantly enhanced the PA imaging intensity (Fig. 3d). The above results proved that AMPG NPs have potential for in vivo MR and PA imaging.

#### In vitro cell culture studies of AMPG NPs

**Fig. 4:**
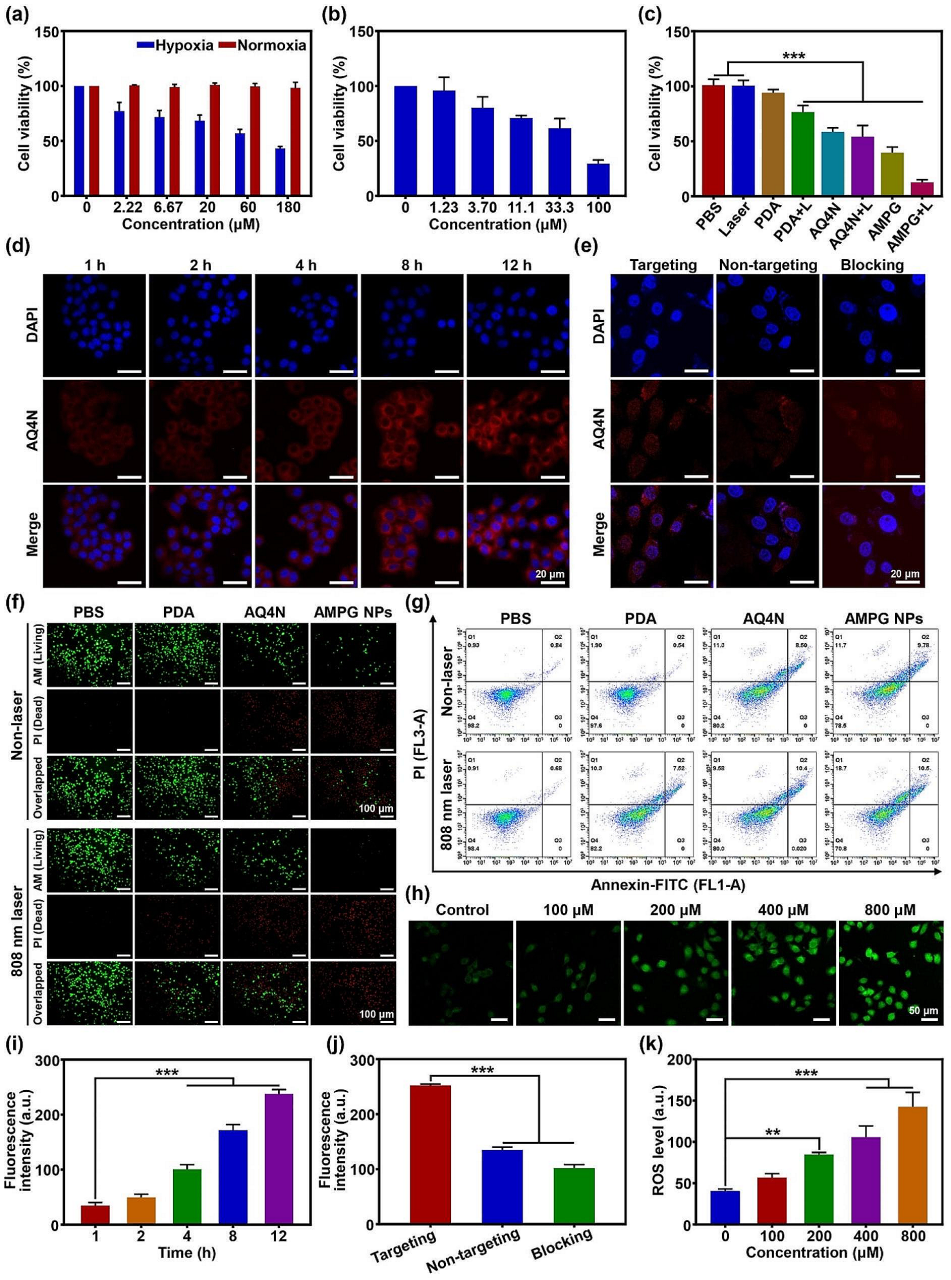
In vitro cell culture studies. **a**) Cell viability to determine the cytotoxicity of different concentrations of AQ4N under normoxic and hypoxic conditions. **b**) Cell viability to determine the cytotoxicity of different concentrations of AMPG NPs under hypoxic conditions. **c**) Cell viability to determine the cytotoxicity of Laser (L), PDA, PDA + L, AQ4N, AQ4N + L, AMPG NPs, and AMPG NPs + L. **d**) Fluorescence images of cell internalization of AMPG NPs at 1, 2, 4, 8 and 12 h; scale bars = 20 μm. **e**) Fluorescence images of targeting, non-targeting, and blocking groups; scale bars = 20 μm. **f**) Fluorescence microscopy images of AM/PI staining living/dead cells treated with PBS, PDA, AQ4N, and AMPG NPs; scale bar = 100 μm. **g**) The effect of PBS, PDA, AQ4N, and AMPG NPs combined with 808 nm laser irradiation on cell viability was analyzed using flow cytometry. **h**) ROS levels in cells incubated with different concentrations of AMPG NPs. **i**) Fluorescence imaging quantitative data of cell internalization of AMPG NPs at 1, 2, 4, 8, and 12 h. **j**) Fluorescence imaging quantitative data of cell internalization of targeting, non-targeting, and blocking groups. **k**) Quantitative analysis of ROS levels in cells incubated with different concentrations of AMPG NPs

The IC_50_ of AQ4N was first examined by assessing cell viability under normoxic and hypoxic conditions using the CCK-8 assay. As shown in Fig. 4a, the concentrations of AQ4N used were 0, 2.22, 6.67, 20, 60, and 180 µM, and all concentrations of AQ4N in a normoxic environment were virtually non-toxic to tumor cells. In a hypoxic condition, cell viability decreased linearly with increasing AQ4N concentration; the survival rate at 180 µM AQ4N was 43.3%, and the IC_50_ was 98 µM. Therefore, subsequent cytotoxicity assays were performed under a hypoxic environment. The cell viability of AMPG NPs is shown in Fig. 4b. Compared with that of free AQ4N, the toxicity of AMPG NPs significantly increased due to the addition of Mn^2+^, and the cell viability was only 29.47% at an AQ4N concentration of 100 µM.

Subsequently, cell viability was evaluated using different materials and under laser irradiation conditions. As shown in Fig. 4c, the percentages of viable cells in the PBS, laser irradiation, and PDA groups were all close to 100%, indicating that the cytotoxicity of laser irradiation alone and PDA was negligible. Compared with the PDA group, the PDA + L group showed some cytotoxicity. However, due to the poor photothermal effect of PDA, it could not kill cells effectively in a short amount of time. The cell survival rates of the AQ4N and AQ4N + L groups were close to 50%, which was consistent with the cytotoxicity of AQ4N in a hypoxic condition. Due to the Fenton-like activity of Mn^2+^, the addition of CDT further decreased cell viability to 39.6% in the AMPG NPs group. Excitingly, cell viability in the AMPG + L group was only 12.9%, suggesting that the combination of PTT/CDT/chemotherapy might be an effective therapeutic strategy to deal with MDR. Figure 4d shows the CLSM images of AMPG NPs internalized by cells after 1, 2, 4, 8, and 12 h of incubation. The cell nuclei labeled with DAPI emitted blue fluorescence, while those labeled with AQ4N emitted red fluorescence. With time, AQ4N gradually accumulated in the cytoplasm, indicating that AMPG NPs could effectively deliver AQ4N into the cells and accumulate in the cytoplasm.

To show the targeting effect of AMPG NPs more clearly, Fig. 4e shows fluorescence images of the targeting, non-targeting, and blocking groups. Compared with that in the non-targeting group, the fluorescence intensity of AQ4N in the targeting group was significantly greater due to the increased expression of the GRP-78 receptor on the cell surface of drug-resistant cells, and GMBP1 could effectively target drug resistance cells overexpressing the GRP-78 receptor. The blocking group was blocked by GMBP1; therefore, the uptake efficiency of AMPG NPs and the intracytoplasmic fluorescence intensity of AQ4N were significantly reduced.

In addition, to investigate the cellular uptake mechanism of the nanoplatform, CLSM imaging was performed after treating the cells with different inhibitors and then co-incubating the nanoplatform with the cells for 0, 2, and 4 h. As shown in Fig. S6a, the AQ4N fluorescence signals of all groups gradually increased with time, but the AQ4N fluorescence intensity of the chloroquine-treated group was significantly lower than that of the other groups. The results of the quantitative analysis of the AQ4N fluorescence intensity in the cytoplasm are presented in Fig. S6b, and the difference in the signal intensity between the chloroquine group and the remaining three groups was highly significant and statistically significant (***P < 0.001). This suggests that the nanoplatform may have entered the cells by internalization mediated by the transferrin-related pathway [40].

Calcein AM/PI fluorescence di-staining and flow cytometry were used to verify the toxicity of the nanoplatform to tumor cells. Figure 4f shows that after being treated in different ways, the living and dead cells in the culture dish emitted different colors of fluorescence by di-staining. Cells in the PBS group emitted predominantly green fluorescence with or without laser irradiation, indicating low cytotoxicity. Cells in the PDA group showed almost no cytotoxicity without laser irradiation but showed slight cytotoxicity when laser irradiation was applied. Laser irradiation had little effect on the cells in the AQ4N group, which exhibited mild cytotoxicity. Compared with AQ4N, AMPG NPs exhibited significantly greater cytotoxicity due to the addition of Mn^2+^, in which red fluorescence began to dominate, exhibiting extreme cytotoxicity after the application of 808 nm laser irradiation to the cells, suggesting that the synergistic effect of hypoxia-activated chemotherapy/CDT/PTT can significantly increase the probability of cell death. As shown in Fig. 4g, we further verified the cytotoxicity of the nanoplatform before and after laser irradiation using flow cytometry. There was slight apoptotic and necrotic cell death in the AQ4N, AMPG NPs, PDA + L and AQ4N + L groups, whereas the number of apoptotic and necrotic cell deaths in the AMPG NPs + L group was significantly greater than that in the other groups. These results also confirmed the results of AM/PI di-staining images, indicating that AMPG NPs combined with laser irradiation could effectively kill tumor cells in vitro.

To further verify the Fenton-like activity of the nanoplatform in vitro, Fig. 4h shows CLSM images of cells co-incubated with different concentrations of AMPG NPs for 4 h and then stained with DCFH-DA. In the control group, only very weak green fluorescence was observed, indicating low ROS levels. However, as the concentration of AMPG NPs increased, the intensity of green fluorescence increased significantly, indicating that the ROS levels increased significantly. Moreover, Fig. S7a shows that the fluorescence intensity of cells co-incubated with AMPG NPs increased over time, suggesting that this nanoplatform co-incubated with tumor cells could generate some ROS in vitro even without adding exogenous H_2_O_2_.

The quantitative data of fluorescence images of cell internalization are shown in Fig. 4i. The content of AQ4N in the cytoplasm increased significantly with increasing incubation time, and the difference between the signal intensity values at 1 h and those at 4, 8, and 12 h was highly statistically significant (***P < 0.001). The quantitative data of fluorescence images of the cell affinity of each group are shown in Fig. 4j, and the signal intensity of the targeting group was significantly greater than that of the non-targeting and blocking groups (***P < 0.001). Figure 4k shows the quantitative data of ROS levels of cells incubated with different concentrations of AMPG NPs. The signal intensities of AMPG NPs (Mn^2+^: 0.4 mM) and AMPG NPs (Mn^2+^: 0.8 mM) were significantly greater than that of the control group (***P < 0.001). Figure S7b shows the quantitative data of ROS levels of cells incubated with AMPG NPs for different durations, and the fluorescence intensity of cells incubated with AMPG NPs increased over time.

### Hemolysis and acute toxicity testing of AMPG NPs

**Fig. 5:**
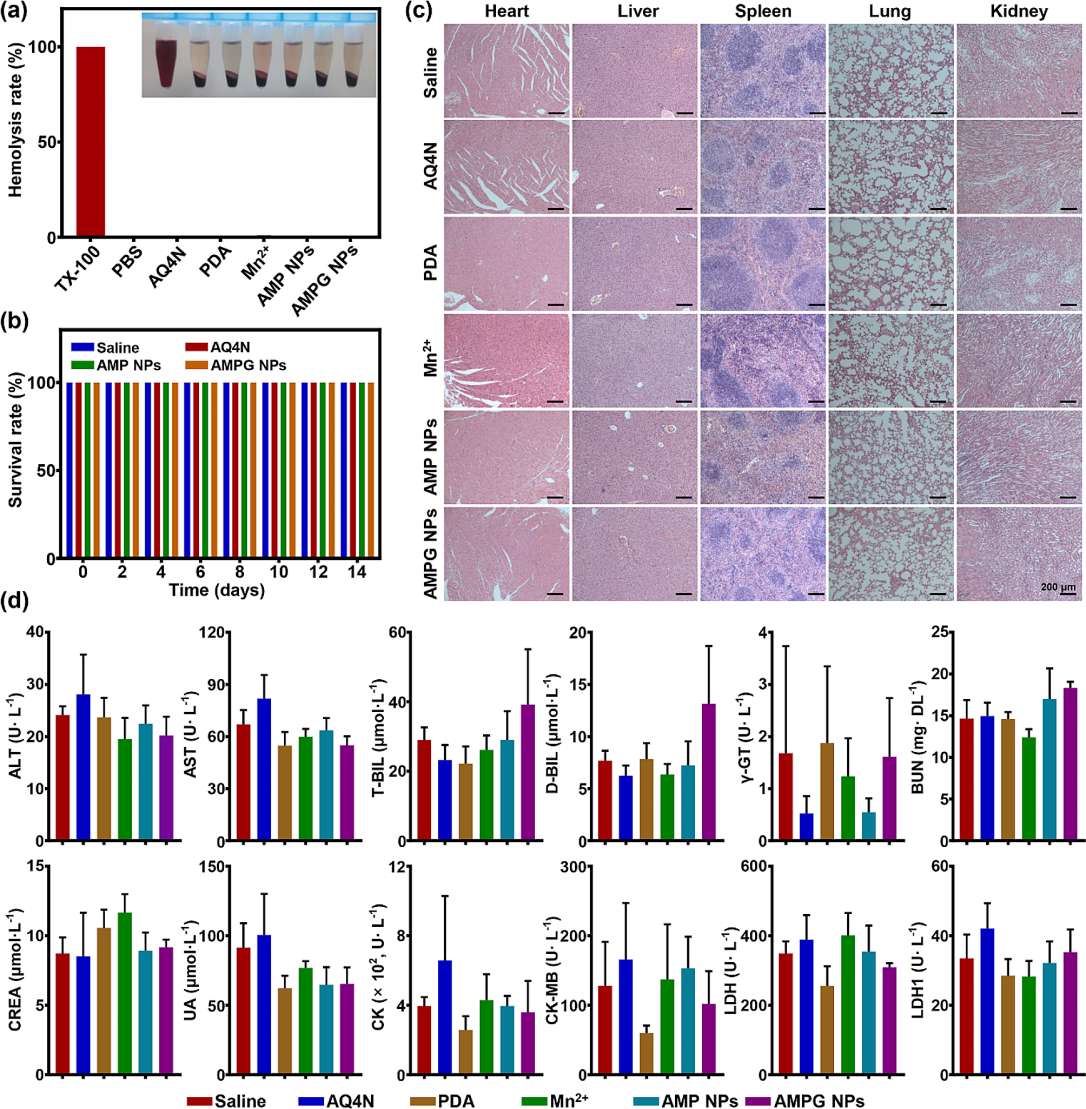
Biosafety of AMPG NPs. **a**) Hemolysis of AQ4N, PDA, Mn^2+^, AMP NPs, and AMPG NPs. Triton X-100 and PBS were positive and negative controls. **b**) Survival of mice in the 14-day acute toxicity test. **c**) H&E-stained tissues from the main organs of mice after the acute toxicity test (heart, liver, spleen, lung, and kidney); scale bars = 200 μm. **d**) Liver, renal function, and cardiac enzyme tests (ALT: alanine transaminase; AST: aspartate aminotransferase; T-BIL: total bilirubin; D-BIL: direct bilirubin; γ-GT: γ-glutamyl transferase; BUN: blood urea nitrogen; CREA: creatinine; UA: uric acid; CK: creatine kinase; LDH: lactate dehydrogenase)

As shown in Fig. 5a, the positive control Triton X-100 group demonstrated significant hemolysis, while the negative control (PBS), AQ4N, PDA, Mn^2+^, AMP NPs, and AMPG NPs groups were all almost free of hemolysis, and the hemolysis rates of the five groups (AQ4N, PDA, Mn^2+^, AMP NPs, and AMPG NPs groups) were quantitatively recorded, which were 0.202%, 0.493%, 0.632%, 0.270% and 0.173%, respectively (see Fig. 5b), indicating that these nanomaterials were fully usable in vivo. Figure 5b shows that during the 14-day acute toxicity test, the behavioral activities of mice in all groups were normal and the survival rate was always 100%. The potential toxicity of the nanoplatform to mice could be further verified by histopathological observation and whole blood biochemical indices. The results of H&E staining and liver, renal function, and cardiac enzyme tests are shown in Fig. 5c and d. Pathological observation showed that the nanomaterials in each group did not cause any damage to the organs and tissues of the mice. The results of blood biochemistry tests were also within the reference range. The above results indicated that the nanomaterials in each group had a high level of animal safety.

### In vivo PA and MR imaging and photothermal performance of AMPG NPs

**Fig. 6:**
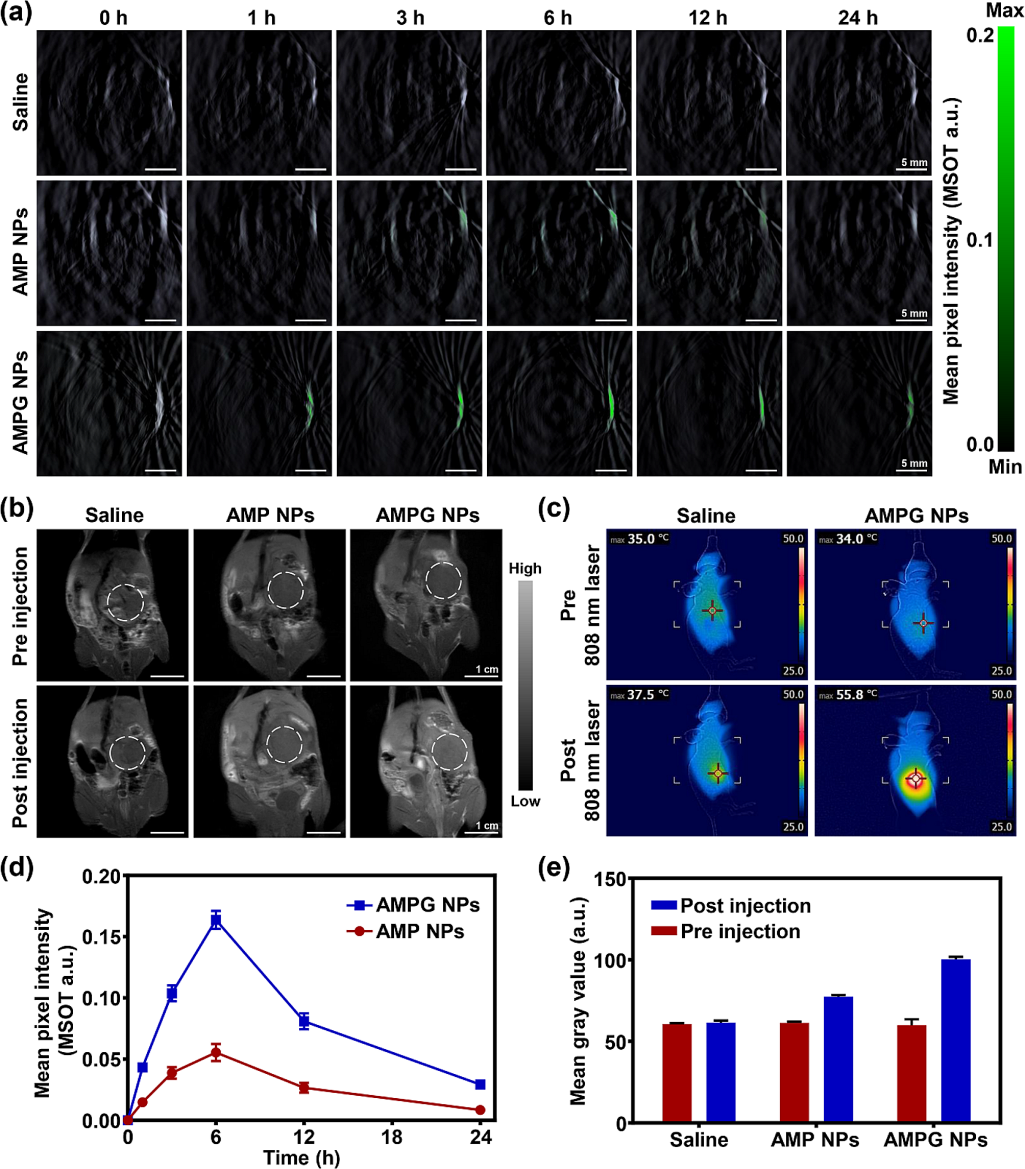
In vivo PA and MR imaging and photothermal effects. **a**) In vivo PA imaging of AMPG NPs targeting tumor-bearing mice; scale bars = 5 mm. **b**) In vivo MR imaging of AMPG NPs targeting orthotopic tumors; scale bars = 1 cm. **c**) NIR thermal images of mice after being treated with PBS and AMPG NPs under pre- and post-irradiation conditions using an 808 nm laser. **d**) Quantitative data of PA imaging at 0, 1, 3, 6, 12 and 24 h. **e**) Quantitative data of MR imaging signal intensity in vivo

Before in vivo therapy, it is necessary to verify whether the nanoplatform can effectively target and be enriched in tumors to achieve in vivo high-temperature PTT. Figure 6a shows the PA images of tumor-bearing mice before treatment and within 24 h after tail vein injection of PBS, AMP NPs, and AMPG NPs, respectively. There was no PA signal throughout the whole process in the PBS group, and the intensity of the PA signal in the tumor areas of the AMP NPs and AMPG MPs groups were both the highest at 6 h and then gradually weakened. Compared with that in the AMP NPs group, the PA signal in the tumor area was stronger in the AMPG NPs group.

Figure 6b shows the MR images of orthotopic tumors in the mouse model before treatment and 6 h after tail vein injection of PBS, AMP NPs, and AMPG NPs, respectively, and the tumor sites are marked with circles. Among them, the signal intensity of the tumor site in the PBS group was almost unchanged, and the signal intensity at the tumor site in the AMP NPs and AMPG NPs groups was enhanced at 6 h after injection; however, the signal intensity at the tumor site in the AMPG NPs group was significantly greater than that in the AMP NPs group.

To verify whether the nanoplatform can effectively achieve high-temperature PTT in the tumor area, it is necessary to carry out an in vivo photothermal performance study of the nanoplatform. As shown in Fig. 6c, before irradiation and after 5 min of 808 nm laser irradiation, the temperature of the PBS group increased from 35℃ to 37.5℃, and the temperature of the AMP NPs group increased from 34℃ to 55.8℃. The in vivo photothermal effect was remarkable, which was significant enough to support subsequent high-temperature PTT in vivo.

The quantification of the PA signal intensity is shown in Fig. 6d using ImageJ. The PA signals of the tumor areas in both groups showed a significant increasing trend from 0 to 6 h and peaked at 6 h. The PA signals of the tumors in the AMPG NPs group were stronger, which indicated that the GMBP1-modified nanoplatform had a greater active targeting ability. The quantification of the grey values of the tumor regions is shown in Fig. 6e, further suggesting that GMBP1 modification significantly enhances the tumor enrichment efficiency of the nanoplatform.

### In vivo synergistic treatment and hypoxia monitoring in tumor-bearing mice

**Fig. 7:**
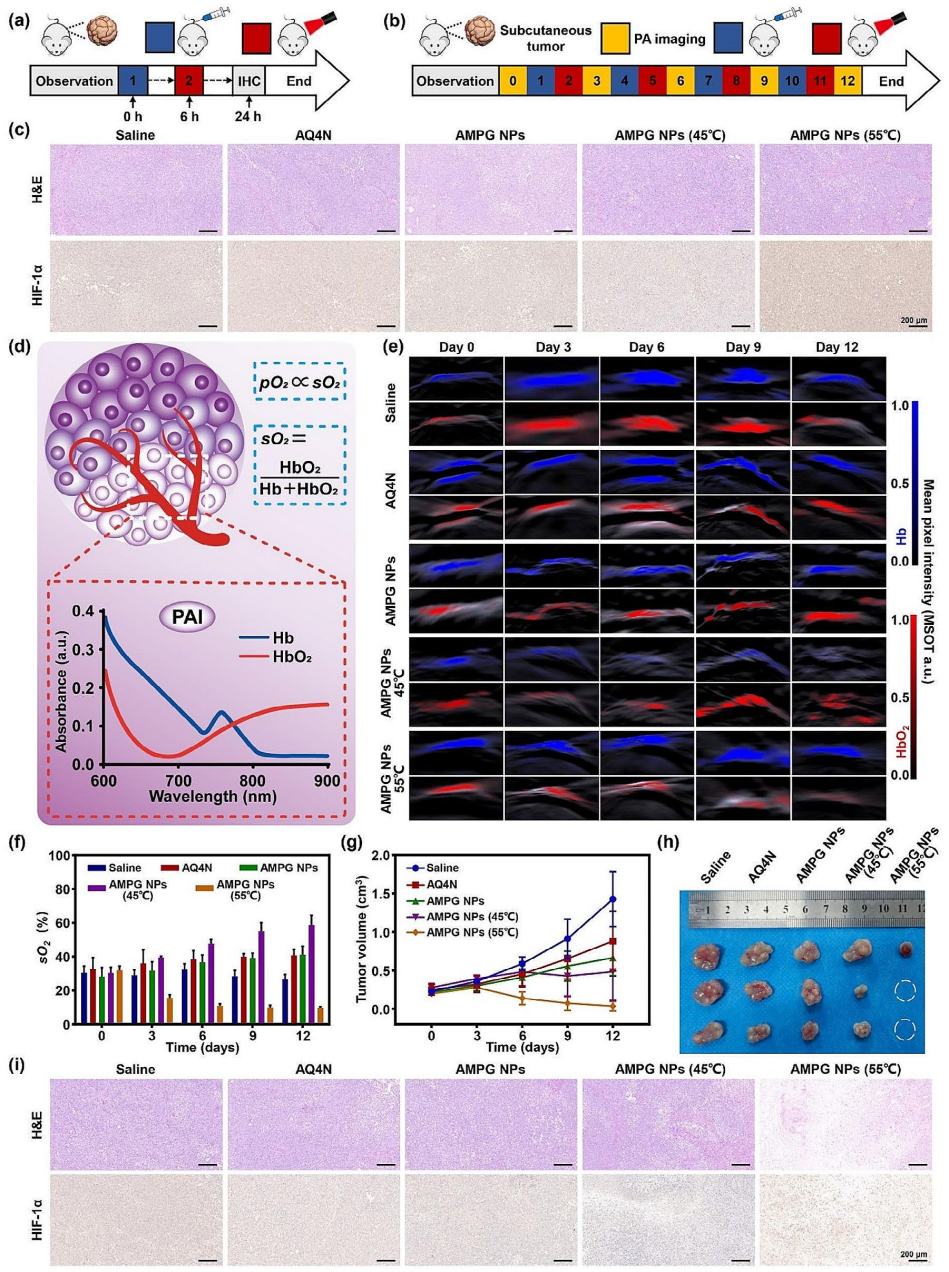
In vivo synergistic treatment and hypoxia monitoring in tumor-bearing mice. **a**) Schematic illustration of IHC validation of high-temperature PTT exacerbating hypoxia. **b**) Schematic illustration of high-temperature PTT exacerbating hypoxia. **c**) IHC staining (HIF-1α) of tumor slices obtained from different treated mice with or without laser irradiation (808 nm) 18 h after injection; scale bars = 200 μm. **d**) Schematic illustration of hypoxia monitoring. **e**) Comparison of PA imaging of Hb/HbO_2_in vivo among the PBS, AQ4N, AMPG NPs, AMPG NPs (45℃) and AMPG NPs (55℃) groups; scale bar = 2 mm. **f**) Quantitative data of *s*O_2_ intensity calculated and shown within 0–12 days. **g**) Quantitative analysis of tumor volumes from days 0 to 12. **h**) Photograph of excised tumors on day 12. **i)** IHC staining (HIF-1α) of tumor slices obtained from mice subjected to different treatments for 12 days; scale bars = 200 μm

To verify that high-temperature PTT can effectively exacerbate the hypoxia status of the tumor area and thus induce hypoxia-activated chemotherapy, we first performed H&E staining and IHC staining analysis of HIF-1α in the tumor sites of mice after different treatments, and the schematic illustration is shown in Fig. 7a. To further investigate the long-term effect of different treatment modalities on the hypoxia status of the tumor sites in mice, we used PA imaging to monitor the blood *s*O_2_ at the tumor sites and analyzed the tumor tissues by H&E and IHC staining again at the end of treatment to facilitate further optimization and determination of the treatment plan, and a schematic illustration is shown in Fig. 7b.

As shown in Fig. 7c, compared with those in the PBS group, there were no significant differences in the hypoxia status of the mice in the AQ4N and AMPG NPs groups and mice in the mild-temperature PTT group with AMPG NPs, while the HIF-1α expression in the AMPG NPs high-temperature PTT group significantly increased, and the degree of hypoxia at the tumor site significantly increased, indicating that high-temperature PTT could exacerbate the level of hypoxia in the tumor microenvironment, which was conducive to enhancing the effect of hypoxia-activated chemotherapy.

A schematic illustration of hypoxia monitoring through PA imaging is shown in Fig. 7d. PA images of Hb and HbO_2_ in the tumor sites of the mice were captured on days 0, 3, 6, 9, and 12, as shown in Fig. 7e. According to the images, the relative levels of Hb and HbO_2_ in the tumor tissues of the PBS group did not change much; the relative levels of Hb in the AQ4N and AMPG NPs groups showed a slight decreasing trend, while the relative levels of HbO_2_ did not change much; the relative levels of Hb and HbO_2_ in the mild-temperature PTT group of AMPG NPs did not change much during the first treatment period, but the relative levels of Hb decreased in the following three treatment periods. Since the partial pressure of oxygen (*p*O_2_) and blood *s*O_2_ are positively correlated, to demonstrate the hypoxia status of the tumor site more intuitively, we calculated the blood *s*O_2_ of the tumor site, as shown in Fig. 7f. By combining the quantitative results of blood *s*O_2_ in the tumor area of each group of mice with the tumor size change curve during the treatment period (Fig. 7g), tumors in the PBS group continued to grow, the hypoxic condition of the tumor site was always at the level of severe hypoxia, and the blood *s*O_2_ was always maintained at approximately 30%. The hypoxic condition of the tumors in the AQ4N and AMPG NPs groups was slightly alleviated, the blood *s*O_2_ was elevated above 40%, and the effect of the tumor treatment was still very poor. In the AMPG NPs mild-temperature PTT group, although the hypoxic condition of the tumor was better relieved and blood *s*O_2_ increased to approximately 60%, the therapeutic effect was still poor, possibly because mild-temperature PTT can relieve the hypoxia of the tumor. However, with the alleviation of hypoxia, the efficacy of AQ4N was significantly decreased, and therefore, AQ4N could not effectively inhibit tumor growth. The hypoxic condition of the tumor in the AMPG NPs high-temperature PTT group significantly worsened after the first treatment cycle, but it was not effective. After four treatment cycles, two of the mice in the AMPG NPs high-temperature PTT group were completely cured of their tumors, and the tumor size of the remaining mice was significantly smaller. This indicates that the use of high-temperature PTT to exacerbate hypoxia in the tumor microenvironment to simultaneously enhance hypoxia-activated chemotherapy and CDT may be an effective synergistic anti-tumor therapy. The anatomical images of the tumors from the mice in each group at the end of treatment are shown in Fig. 7h. In addition, as shown in Fig. 7i, we performed H&E staining and IHC analysis on the tumors, which again confirmed that high-temperature PTT effectively exacerbated the hypoxia at the tumor site. Therefore, we confirmed that high-temperature PTT combined with dual-enhanced hypoxia-activated chemotherapy and CDT was an effective synergistic treatment modality.

### In vivo high-temperature PTT/hypoxia-activated chemotherapy/CDT synergistic treatment

**Fig. 8:**
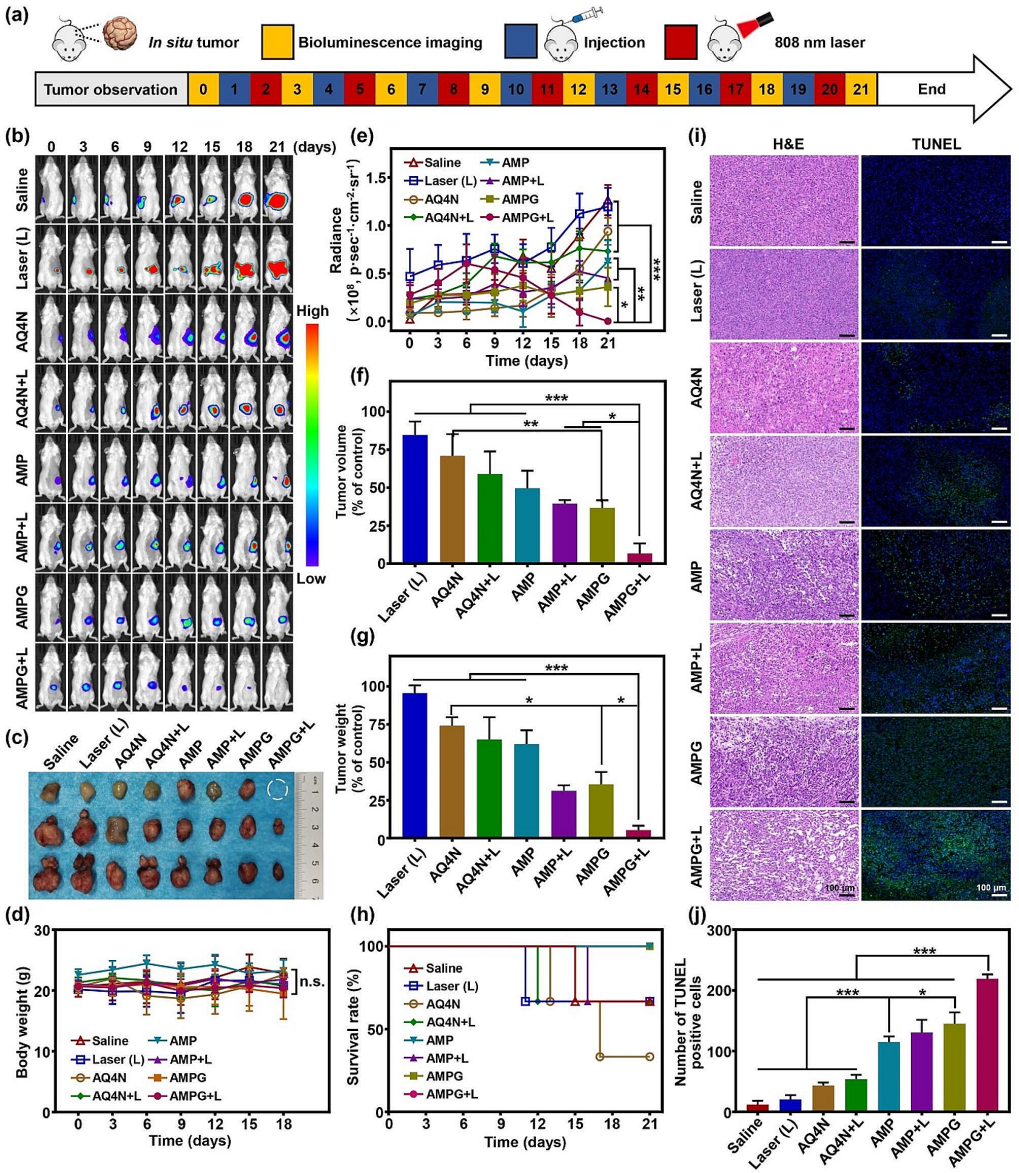
In vivo synergistic high-temperature PTT/hypoxia-activated chemotherapy/CDT. **a**) Schematic illustration of synergistic orthotopic drug-resistant tumor therapy. **b**) In vivo bioluminescence imaging of orthotopic gliomas from days 0 to 15. **c**) Photograph of the excised tumor on day 21. **d**) Quantitative data of body weights of mice measured from days 0 to 18. **e**) Quantitative intensity of bioluminescence imaging from days 0 to 21. **f**) Quantitative data of relative volume of tumors compared to control tumors on day 21. **g**) Quantitative data of relative weight of tumors compared to control tumors on day 21. **h**) Percent survival of mice in the PBS, Laser (L), AQ4N, AQ4N + L, AMP NPs, AMP NPs + L, AMPG NPs, and AMPG NPs + L groups. **i**) H&E and TUNEL staining were used to determine tumor apoptosis; scale bars = 100 μm. **j**) Quantitative data of TUNEL staining

Figure 8a shows a schematic diagram of our design to study the anti-tumor effects in mice with orthotopic tumors. Tumor size was monitored using bioluminescence at regular intervals after tumor inoculation, and different treatments were administered to each group of mice when their tumors grew to the appropriate size. Bioluminescence imaging was started on day 0, the nanoplatform was injected at 18:00 on the first day, and laser irradiation was applied to the laser irradiation group at 0:00 on the second day, with a cycle lasting 3 days. The efficacy of high-temperature combined PTT with dual-enhanced hypoxia-activated chemotherapy and CDT was assessed by a 21-day in vivo antitumor study.

The bioluminescence images of the mice in each group throughout the treatment are shown in Fig. 8b. The tumors of the mice in the PBS group and Laser (L) group grew rapidly, with increasing bioluminescence, and then spread to almost the whole abdominal cavity. The antitumor effect was also poor in the AQ4N and AQ4N + L groups due to a complete lack of targeting of the drug, the high toxicity of AQ4N only in the area of hypoxia, and the lack of good properties of AQ4N alone. Both AMP NPs and AMP NPs + L showed some tumor inhibitory effects, but the therapeutic effect was not prominent because they could only target tumor tissues passively. Compared to the other groups, AMPG NPs + L group showed an excellent anti-tumor effect, with the bioluminescence signal becoming increasingly weaker as the treatment progressed since day 9, and the signal was eventually eliminated. Figure 8c shows the final tumor volume, which was consistent with the above results, except for the saline group and laser irradiation group; all other groups showed some tumor suppression effects. However, only the AMPG NPs + L group showed the best therapeutic effect, in which the tumors of one of the mice were completely ablated, and the tumor volume of the remaining two mice was also significantly smaller than that of the other treatment groups, implying that dual-enhanced hypoxia-activated chemotherapy and CDT using high-temperature PTT achieved by AMPG NPs was an extremely effective antitumor tool. The body weights of the mice in each group were recorded every three days during the entire treatment period. As shown in Fig. 8d, the body weights of the mice in each group were close to each other during the treatment period, indicating that the nanomaterials in each group had good biocompatibility.

The quantitative results of the bioluminescence signal intensity of each group of mice during the treatment period are shown in Fig. 8e. The bioluminescence signal intensities of the PBS, Laser (L), AQ4N, and AQ4N + L groups generally tended to gradually increase over time. The bioluminescence signals of the AMP NPs group, AMP NPs + L group, and AMPG NPs group showed a slow increase, demonstrating a certain tumor inhibitory effect. The bioluminescence signal intensity in the AMPG NPs + L group showed an increasing trend and then a decreasing trend, which eventually disappeared completely, indicating that high-temperature PTT with simultaneous enhancement of hypoxia-activated chemotherapy and CDT was an effective synergistic treatment.

Since it was not possible to measure the size of in situ tumors in real-time during treatment, the tumor volumes were measured by euthanizing and dissecting the mice at the end of treatment (Fig. 8f). The tumor volumes in the PBS and Laser (L) groups were very large, and the growth of the tumors was not inhibited. Compared with the PBS and Laser (L) groups, the AQ4N, AQ4N + L, AMP NPs, AMP NPs + L, and AMPG NPs groups showed a decrease in tumor volume, but still failed to exhibit inhibited tumor growth. Due to its excellent targeting performance, the AMPG NPs + L group could effectively achieve high-temperature PTT, which not only exacerbated hypoxia and enhanced the efficacy of hypoxia-activated chemotherapy but also significantly improved the efficiency of ·OH generation in the tumor site, with a remarkable anti-tumor effect. Statistical analysis showed that the AMPG NPs + L group was significantly different from the AMP NPs + L and AMPG NPs groups in terms of tumor volume inhibition (*P < 0.05) and was extremely significantly different from the other groups (***P < 0.001). In addition, the results of the tumor weight test are shown in Fig. 8g, which was in general agreement with the results shown in Fig. 8c and f.

Figure 8h shows the real-time survival rate of mice in each group during the treatment period, with one mouse in the PBS group dying on day 19, one mouse in the Laser (L) group dying on day 11, two mice in the AQ4N group dying on day 13 and day 17, one mouse in the AQ4N + L group dying on day 12, and one mouse in the AMP + L group dying on day 16; the AMP NPs, AMPG NPs, and AMPG NPs + L groups did not experience any mouse death during the treatment period, indicating that these treatments could effectively improve the survival rate of orthotopic drug-resistant tumors.

### Tissue section image analysis

Mice in each group were euthanized after 21 days of treatment, and the tumor tissues were observed and analyzed via pathological sections. As shown in Fig. 8i, only the tumor tissues of mice in the PBS and Laser (L) groups were barely damaged, and the tumor cell morphology remained normal. The tumor tissues of the remaining treatment groups were damaged to some extent, but those of the AMPG NPs + L group were damaged to the greatest extent. In addition, the results of the TUNEL fluorescence staining analysis are shown in Fig. 8i, where there were almost no green fluorescent areas in the PBS and Laser (L) groups, and no apoptosis was detected. Some green fluorescent areas appeared in the AQ4N group, the AQ4N + L group, AMP NPs group, AMP NPs + L group, and AMPG NPs group, but the percentage of the total amount of green fluorescent area was still not obvious. In contrast, the AMPG NPs + L group showed strong green fluorescence with a great percentage of the area, and this was proven by the quantitative data of TUNEL staining (Fig. 8j), indicating that the use of this nanoplatform to achieve high-temperature PTT dual-enhanced hypoxia-activated chemotherapy and CDT could effectively achieve synergistic treatment of in situ drug-resistant tumors.

### Conclusion

In conclusion, we designed AMPG NPs and effectively improved the efficiency of AMPG NPs in actively targeting drug-resistant tumors via GMBP1 modification. Both cell culture and animal experiments indicated that AMPG NPs combined with laser irradiation had a significant inhibitory effect on drug-resistant tumors. This effect results from the excellent photothermal conversion ability of AMPG NPs, which could achieve local high-temperature PTT (> 50℃) in tumor tissues, exacerbating the hypoxia of the microenvironment to enhance hypoxia-activated chemotherapy and directly enhancing the efficiency of ·OH generation. Tissue section studies and PA imaging hypoxia monitoring during treatment demonstrated that high-temperature PTT could effectively exacerbate the hypoxic conditions at the tumor site, improve the chemotherapeutic efficacy of AQ4N, and significantly increase the apoptosis of drug-resistant cells without causing adverse effects on normal mice. These findings suggest that AMPG NPs and their dual enhancement of hypoxia-activated chemotherapy and CDT by high-temperature PTT are promising synergistic therapeutic options for the treatment of drug-resistant cancers and are expected to be useful for treating other tumors.

### Electronic supplementary material

Below is the link to the electronic supplementary material.

Supplementary Material 1

## Data Availability

No datasets were generated or analysed during the current study.

## Abbreviations

MDR: Multidrug resistance
PDT: Photodynamic therapy
PTT: Photothermal therapy
NIR: Near-infrared
CDT: Chemodynamic therapy
·OH: Hydroxyl radicals
PA: Photoacoustic
PCE: Photothermal conversion efficiency
AMPG NPs: AQ4N-Mn(II)@PDA-GMBP1
AMP NPs: AQ4N-Mn(II)@PDA NPs
MR: Magnetic resonance
IHC: Immunohistochemistry
EDS: Energy-dispersive spectroscopy
XPS: X-ray photoelectron spectroscopy
TEM: Transmission electron microscopy
DLS: Dynamic light scattering
FTIR: Fourier transform infrared
FBS: Fetal bovine serum
DI water: Deionized water
DEE: Drug encapsulation efficiency
DLC: Drug loading content
MB: Methylene blue
CLSM: Confocal laser scanning microscopy
TUNEL: Terminal deoxynucleotidyl transferase dUTP-biotin nick ends labeling
