# Supplementary material for: High-temperature PTT/CDT coordination nanoplatform realizing exacerbated hypoxia for enhancing hypoxia-activated chemotherapy to overcome tumor drug resistance
Source: J Nanobiotechnology. 2024 Jun 26;22:374. doi: 10.1186/s12951-024-02653-8 (PMC11200845; doi:10.1186/s12951-024-02653-8)
Supplement: Supplementary file 1 — Supplementary Material 1 [file 12951_2024_2653_MOESM1_ESM.docx]

**Supporting information**

High-temperature PTT/CDT coordination nanoplatform realizing exacerbated hypoxia for enhancing hypoxia-activated chemotherapy to overcome tumor drug resistance

Peng Chang^1^, Yingying Guo^2^, Dan Chen^1^, Ke Li^3^, Wei Wang^4^, Zhihua Yang^4^, Jingwen Ma^5^, Yun Zeng^1*^, Wenhua Zhan^4*^, and Yonghua Zhan^1*^

^1^ School of Life Science and Technology, Xidian University and Engineering Research Center of Molecular and Neuro Imaging, Ministry of Education, Xi’an 710126, PR China

^2^ Institute of Analytical Chemistry and Instrument for Life Science. School of Life Science and Technology, Xian Jiaotong University, Xi’an 710049, PR China

^3^ Xi’an Key Laboratory for Prevention and Treatment of Common Aging Diseases, Translational and Research Centre for Prevention and Therapy of Chronic Disease, Institute of Basic and Translational Medicine, Xi’an Medical University, Xi’an 710021, PR China

^4^ Department of Radiation Oncology, General Hospital of Ningxia Medical University, Yinchuan 750004, PR China

^5^ Radiology Department, CT and MRI Room, Ninth Hospital of Xi’an, Xi’an 710054, PR China

Corresponding authors

Yonghua Zhan - School of Life Science and Technology, Xidian University and Engineering Research Center of Molecular and Neuro Imaging, Ministry of Education, Xi’an 710126, PR China; Email: yhzhan@xidian.edu.cn

Wenhua Zhan - Department of Radiation Oncology, General Hospital of Ningxia Medical University, Yinchuan 750004, PR China; Email: zhanwhgood@163.com

Yun Zeng - School of Life Science and Technology, Xidian University and Engineering Research Center of Molecular and Neuro Imaging, Ministry of Education, Xi’an 710126, PR China; Email: yzeng@xidian.edu.cn

Jingwen Ma - Radiology Department, CT and MRI Room, Ninth Hospital of Xi’an, Xi’an 710054, PR China; Email: majingwen891031@xjtu.edu.cn

**1. Chemical and consumables**

MnAc_2_ was purchased from Merck KGaA, Darmstadt (Germany). Dopamine hydrochloride, Tris-HCl, and methylene blue (MB) were purchased from Shanghai Aladdin (China). AQ4N was purchased from MedChemexpress Bio-Technology (USA). Sheep red blood cells (4%) were purchased from Shanghai Yuanye Bio-Technology (China). GMBP1 was synthesized by Shanghai GL Biochemical Technology (China) (shown in Figure S1). All cell lines were provided by Xi’an Medical University. Fetal bovine serum (FBS), Trypsin, DMEM cell culture medium, and Triton X-100 were purchased from Thermo Fisher Scientific (China). DAPI was purchased from Wuhan Boster Biological Technology (China). CCK-8 kit was purchased from Dojindo Molecular Technologies (USA). The Annexin V-FITC/PI cell apoptosis detection kit, Reactive oxygen species assay kit, and 4% paraformaldehyde were purchased from Beijing Labgic (Biosharp) Science & Technology (China). Calcein AM/PI cell viability/cytotoxicity assay kit was purchased from Shanghai Beyotime Biotechnology (China). Cell culture consumables were purchased from Corning Incorporated (USA). D-Luciferin potassium salt was purchased from GlpBIO Technology (USA). The H&E staining kit, anti-HIF-1α antibodies, and TUNEL apoptosis detection kit were purchased from Wuhan Servicebio Technology (China). BALB/c white and nude mice were purchased from Chongqing Tengxin Biotechnology (China). All chemicals were used directly without further purification.

**2. Instruments**

| Names | Models | Manufacturers | Countries |
| --- | --- | --- | --- |
| UV-vis spectrophotometer | Evolution 201 | Thermo Fisher Scientific | USA |
| Ultrasonic cleaner | KQ3200DE | Kunshan Ultrasonic Instruments | China |
| High-speed centrifuge | Sorvall ST16 | Thermo Fisher Scientific | USA |
| TEM | Talos L120C | Thermo Fisher Scientific | USA |
| 808 nm multimode fiber-coupled lasers | MW-GX-808 | Changchun Laser Optoelectronics Technology | China |
| Handheld NIR thermal camera | E4 | Teledyne FLIR | USA |
| PA imaging system | MSOT inVision128 | iTheraMedical | Germany |
| Small animal living imaging system | IVIS Lumina Series III | PerkinElmer | USA |
| Microplate reader | Infinite 200 Pro | Tecan | Switzerland |
| Confocal laser scanning microscopy (CLSM) | TCS SP5 II | Leica | Germany |
| Fluorescence microscopy | Eclipse Ti-S | Nikon | Japan |
| Flow cytometer | Flow cytometer | BD | Australia |
| Microtome | Jung CM3000 | Leica | Germany |

**3.** **Studies on mechanisms of cellular internalization**

The common cellular internalization pathways are macropinocytosis, clathrin, transferrin-mediated internalization, and caveolae-mediated endocytosis, which can be blocked by amiloride, chlorpromazine, chloroquine, and mycobacteria, respectively. When the cells were attached to the wall and grew to occupy the area of dishes about 70%, the old medium was discarded and the cells were washed three times with PBS, and serum-free medium containing different inhibitors (amiloride, mycophenolate, chlorpromazine, chloroquine) was added. After a total of 2 h of incubation, 1 mL of serum-free medium containing AMP NPs was added and they were incubated for 0.5, 2, and 4 h. The cells were subsequently fixed with 4% paraformaldehyde and stained with DAPI. Cellular internalization inhibition was observed by the CLSM. The quantitative analysis of cellular fluorescence intensity was performed by ImageJ.

**4.** **Animal model construction**

All animal experiments were performed in accordance with the animal ethics of the affiliated institutions (No. KYLL-2021-609). The SGC-7901/ADR-Luc cell line was used for tumor-bearing and orthotopic tumor mouse model, respectively. The tumor-bearing mouse model were established by subcutaneous injection of the cell suspension (5 × 10^6^ cells, 100 µL) into the dorsal side of the mice, and the orthotopic tumor mouse model was established by surgical injection of the cell suspension (5 × 10^6^ cells, 100 µL) into the gastric lining of the mice. BALB/c nude mice (female, 14-16 g, approximately 4 weeks) for tumor-bearing mice, and BALB/c mice (female, 15-17 g, approximately 4 weeks) for orthotopic tumor mouse model.

**5.** **Hemolysis, acute toxicity test, and blood biochemical analysis**

4% sheep blood erythrocytes were used for the hemolysis assay, 1% Triton X-100 solution was set as a positive control, and PBS was set as a negative control. 20 µL of each sample (1% Triton X-100, PBS, AQ4N, Mn^2+^, AMP NPs, and AMPG NPs), 480 µL of saline, and 500 µL of 4% sheep’s blood erythrocyte suspension were added to a 1.5 mL centrifuge tube, which were incubated in a 37°C thermostat for 2 h. After the incubation, these tubes were centrifuged at 10,000 × g for 5 min, and then 200 µL of supernatant of each group was pipetted slowly and carefully into a 96-well plate, and the absorbance at 545 nm was measured using a microplate reader. The hemolysis rates of different samples were calculated according to the following equation:

$$Hemolysis rate (\%)=\frac{{OD}_{S}-{OD}_{N}}{{OD}_{P}-{OD}_{N}}\times100\%$$

*OD_N_* indicates the absorbance at 545 nm of PBS, *OD_P_* indicates the absorbance at 545 nm after treatment with 1% Triton X-100, and *OD_S_* indicates the absorbance at 545 nm after treatment with different samples.

Eighteen BALB/c mice (15-17 g, approximately 4 weeks) were randomly divided into six groups, and each group was injected i.v. with 200 µL of PBS, AQ4N, AMP NPs, and AMPG NPs (dosages: 0.2 mM AQ4N, 0.8 mM Mn^2+^, and 0.4 mg·mL^-1^ PDA), and the mice were observed to survive over 14 days and body weights were measured every two days. After euthanasia of the mice after 14 days, liver and kidney functions as well as cardiac enzymes were examined using blood biochemistry analysis, and in addition, the hearts, livers, spleens, lungs, and kidneys of the euthanized mice were collected for H&E staining.

**6.** **Equations S1-S4 are included in experimental section**

Drug encapsulation efficiency (DEE) and drug loading content (DLC):

$$\mathrm{DEE}\left( wt\% \right)=\frac{\mathrm{mass}of AQ4N input-mass of free AQ4N}{\mathrm{mass}of AQ4N input} (S1)$$

$$\mathrm{DLC}\left( wt\% \right)=\frac{\mathrm{mass}of AQ4N input-mass of free AQ4N}{\mathrm{mass}of AMPG NPs} (S2)$$

PCE:

$$\eta=\frac{hA\Delta T-Q_{S}}{I(1-{10}^{-\lambda})} (S3)$$

where ℎ is the heat transfer coefficient; 𝐴 is the laser irradiation area; Δ𝑇 is the increased temperature; 𝑄*_S_* is the thermal energy of quartz cuvette and solution absorbed from light; 𝐼 is the laser power; 𝜆 is the absorbance at 808 nm.

Tumor volume:

$$V=\frac{1}{2}\times a{\times b}^{2} (S4)$$

where $a$ is the long diameter and $b$ is the short diameter.

**7. Supplementary Figures S1-S7 are included**





**Figure S1.** Design and modification of GMBP1 (GMBP1, which can effectively target drug resistance cells overexpressing the GRP-78 receptor).


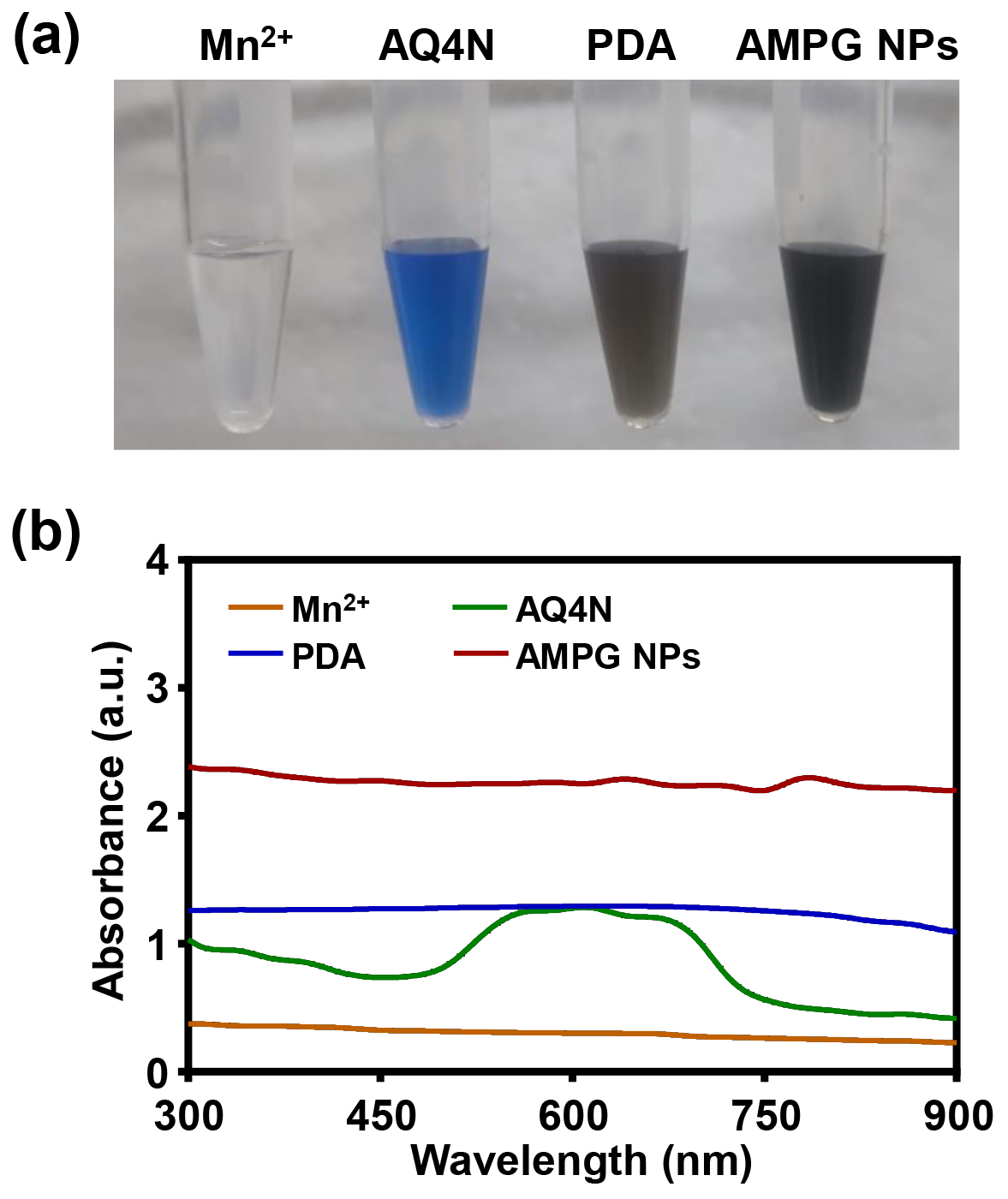


**Figure S2.** a) Photograph of Mn^2+^, AQ4N, PDA, and AMPG NPs. b) UV-vis-NIR spectra of Mn^2+^, AQ4N, PDA, and AMPG NPs.


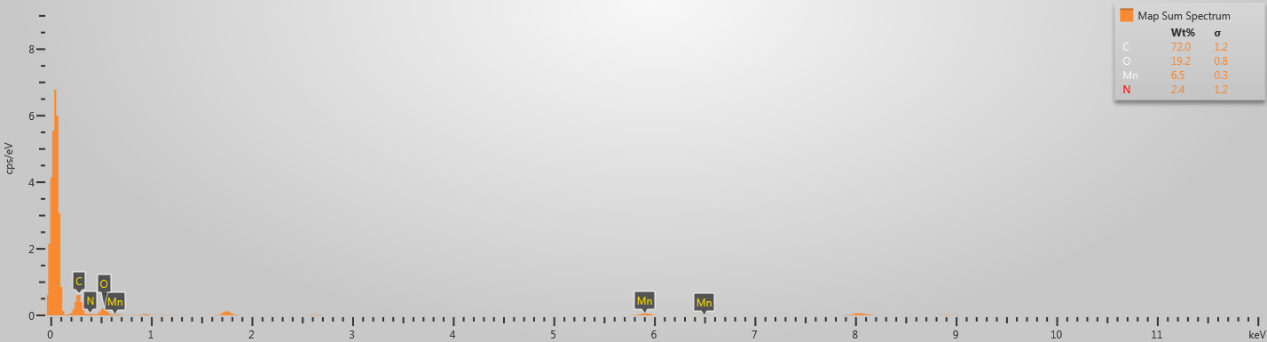


**Figure S3.** Summarized EDS spectrum of AMPG NPs.


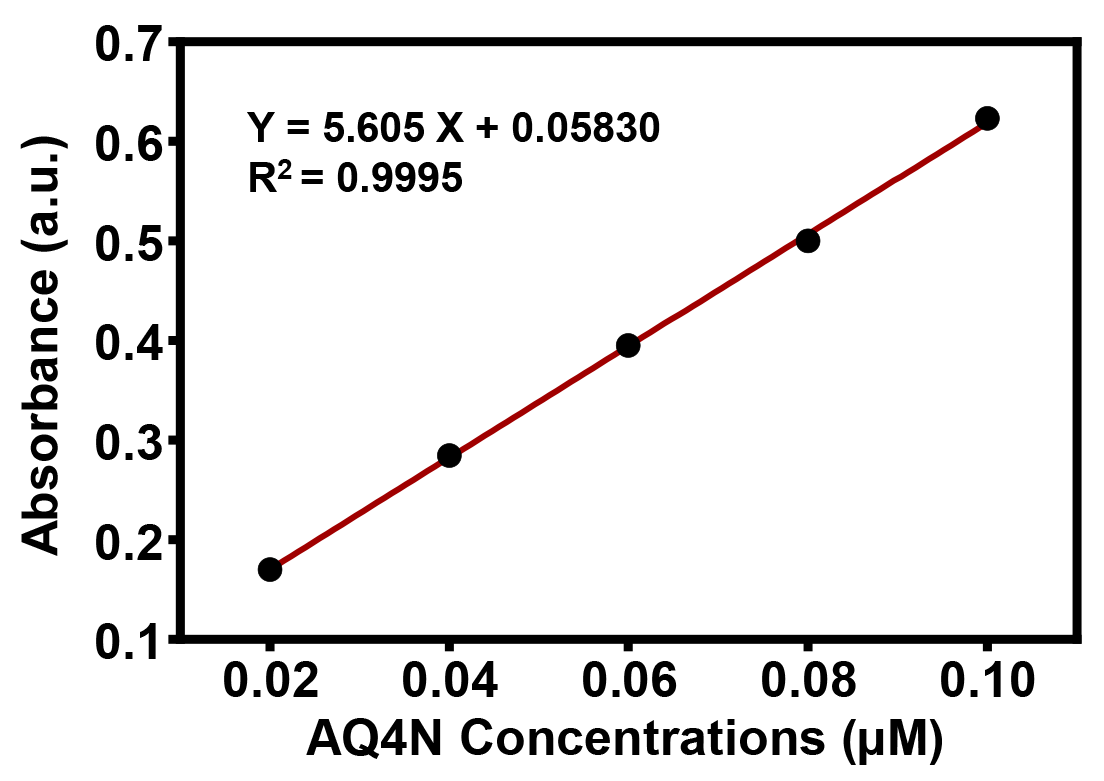


**Figure S4.** Standard curve of AQ4N.


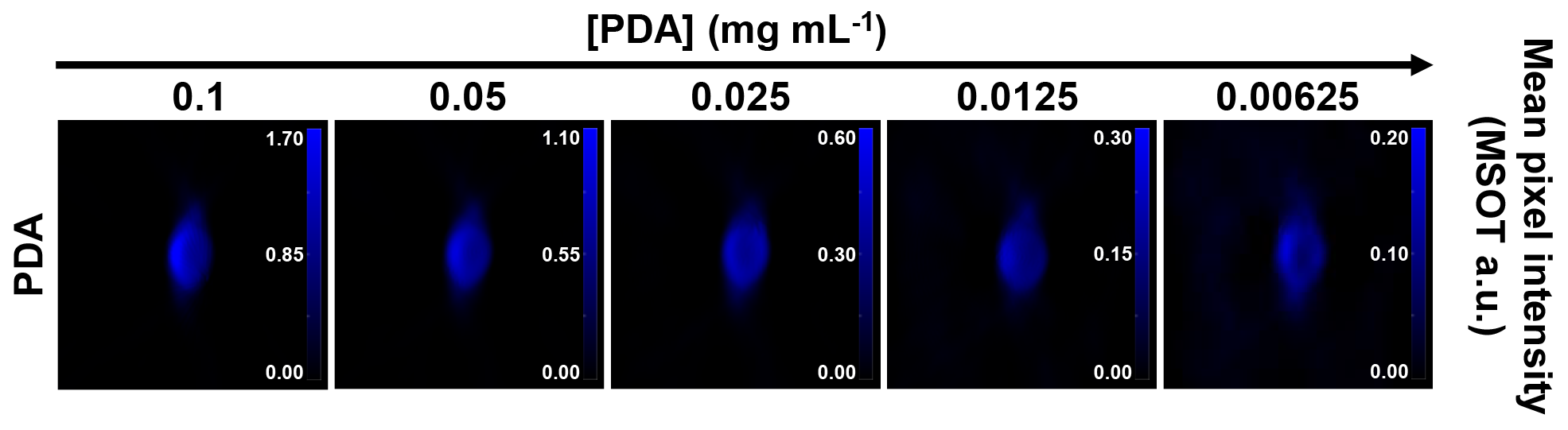


**Figure S5.** PA images of PDA with different concentrations of 0.00625, 0.0125, 0.025, 0.05, and 0.1 mg·mL^-1^ in phantom.


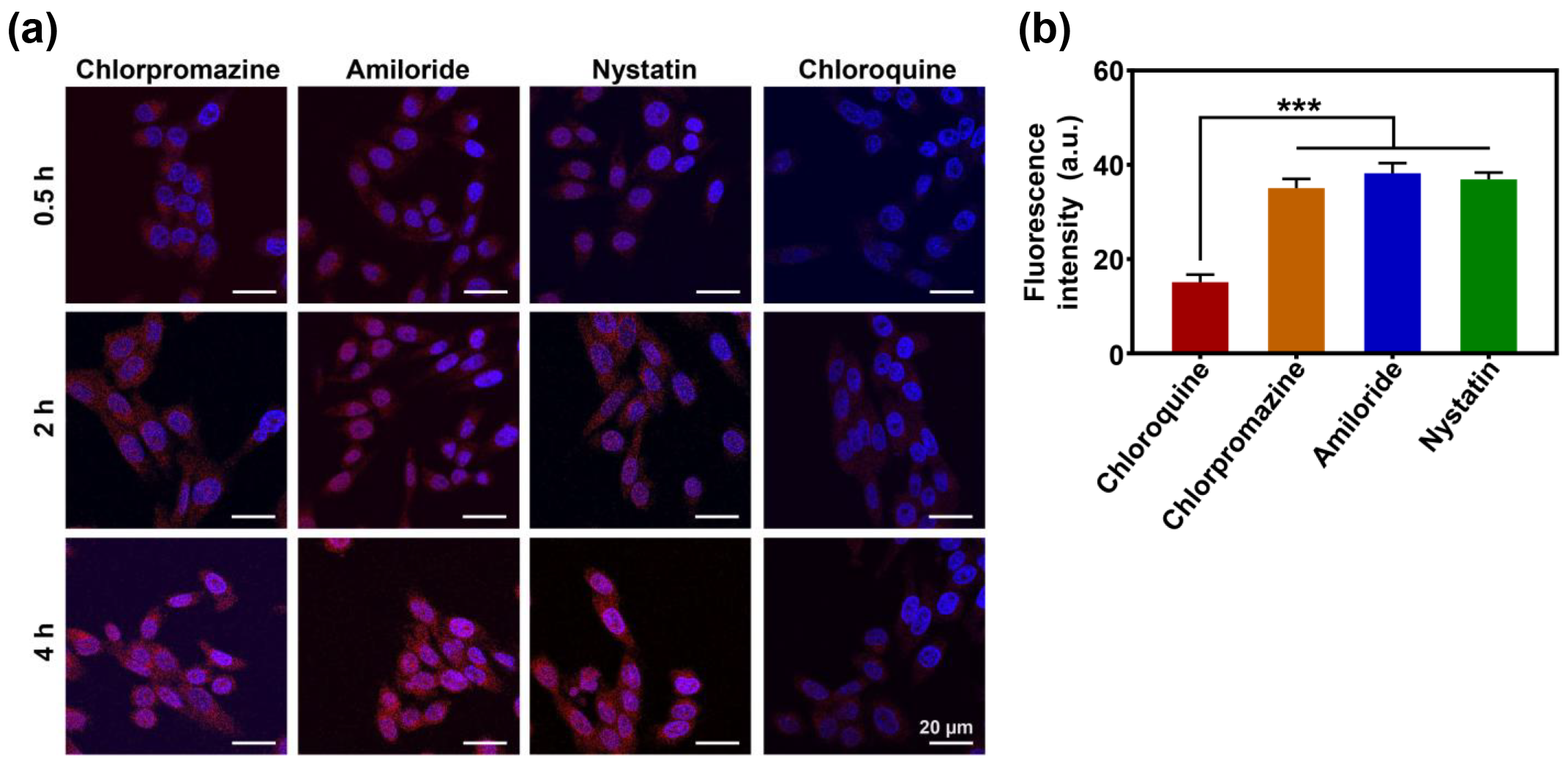


**Figure S6.** Studies on the mechanism of cell internalization of AMPG NPs. a) Fluorescence imaging of cell internalization of AMPG NPs under different conditions (Chlorpromazine, amiloride, nystatin, and chloroquine), scale bars = 20 μm. b) Fluorescence imaging quantitative data of cell internalization of AMPG NPs under different conditions.


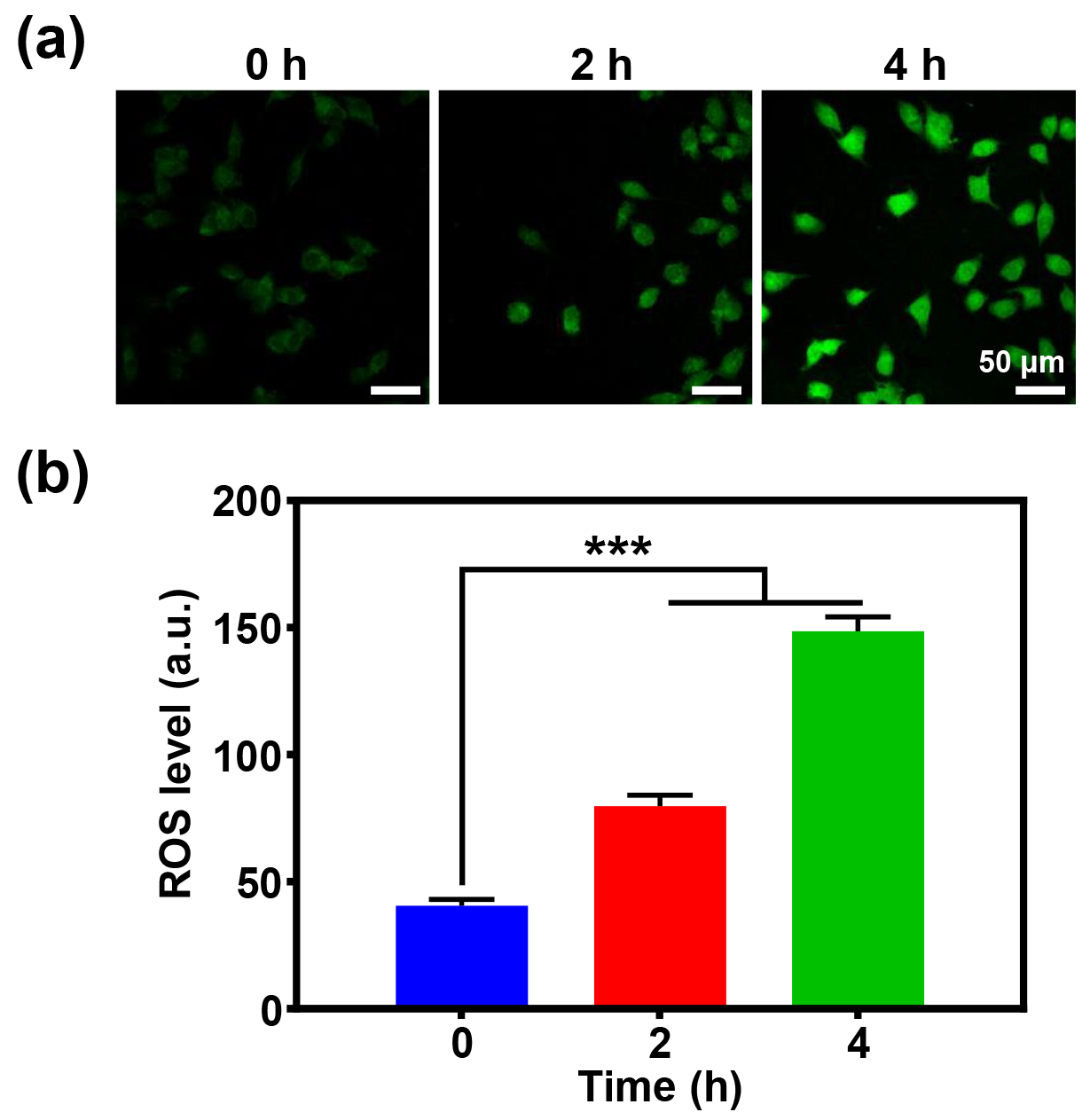


**Figure S7.** ROS levels of cells incubated with AMPG NPs at different times. a) Fluorescence images of ROS levels of cells incubated with AMPG NPs at 0, 2, and 4 h, scale bars = 50 μm. b) ROS levels quantitative data of cells incubated with AMPG NPs at 0, 2, and 4 h.
